# Neonatal mortality and its determinates in public hospitals of Gamo and Gofa zones, southern Ethiopia: prospective follow up study

**DOI:** 10.1186/s12887-019-1881-0

**Published:** 2019-12-16

**Authors:** Abera Mersha, Agegnehu Bante, Shitaye Shibiru

**Affiliations:** Department of Nursing, College of Medicine and Health Sciences, Arba Minch University, Arba Minch, Ethiopia

**Keywords:** Neonatal mortality, Neonatal deaths, Gamo and Gofa zones

## Abstract

**Background:**

The neonatal period is the most vulnerable time for child survival. The declines in the neonatal mortality rate have been slower than the post-neonatal under-five mortality rate in the majority of countries. This trend is also similar in Ethiopia, that neonatal mortality was high as compared to the post-neonatal mortality rate. A large proportion of neonatal deaths occur during the 48 h after delivery. Different studies were conducted in assessing determinates for neonatal mortality but there is a need to assess the immediate postnatal (within 2 days following delivery) cause of neonatal mortality that the majority of deaths occurred at that time. So, this study is to fill those gaps of the aforementioned studies, in assessing the determinate factors affecting neonatal mortality in public hospitals of Gamo and Gofa Zones, Southern Ethiopia.

**Methods:**

A prospective follow up study was conducted among 6769 study participants from April 5, 2018, to March 5, 2019. All live births at the hospitals during the study period were included in this study. A structured verbal autopsy questionnaire was used to collect the data on the causes of neonatal death. Data were entered into Epi data version 3.1 and exported to Stata version 15 for analysis. Crude and adjusted estimate β with 95%CI was calculated in the binary logistic regression model. A log-likelihood ratio (LR) was tested for goodness of fit. In this study *P*-value < 0.05 was considered to declare a result as a statistically significant association.

**Results:**

In this study, neonatal mortality incidence ratio was 9.6 (95%CI: 7.5, 12.2) per 1000 live births. Age of the mother, number of antenatal care visits, sex of the neonate, presentation, and gestational age were identified as the significant determinates for neonatal mortality cases. Prematurity, infection, and birth asphyxia were the most common causes of neonatal mortality cases.

**Conclusions:**

This study indicated that a significant number of neonates died during the neonatal period. Both maternal and neonatal factors were identified. Therefore, early identification of obstetric complications and immediate interventions, strengthening the provision of quality antenatal and postnatal care services are recommended.

## Background

Globally, there is impressive advancements have been made on many health fronts from 2000 to 2017. However, to meet the Sustainable Development Goals’ health targets by 2030, progress must be accelerated, in particular regions with the highest burden of disease [1]. The third Sustainable Development Goals (SDG3) aimed to end preventable deaths of newborns and reduce neonatal mortality to at least as low as 12 per 1000 live births in all countries [2].

Despite all efforts to decrease neonatal mortality, recent data show that neonatal mortality has declined at a slower rate than overall childhood mortality, which has resulted in neonatal mortality now accounting for 46% of overall under-five childhood deaths [3]. The neonatal mortality is 18 globally, and 26.7 in Africa in 2017, and 30 in Ethiopia per 1000 live births in 2019 [4, 5]. Ethiopia Mini-Demographic Health Survey, 2019 indicated that there is a slight increase in neonatal mortality, and it was high as compare to the post-neonatal mortality rate. A large proportion of neonatal deaths occur during the 48 h after delivery, and these first 2 days following delivery are critical for monitoring complications arising from the delivery [4, 6].

World Health Organization (WHO) recommended that after uncomplicated spontaneous vaginal delivery in the health care institution, both the mother and newborn should receive care for 24 h. For home delivery, both the mother and newborn must receive postnatal examination as soon as possible within 24 h if occur at home [7, 8].

The period around birth constitutes a critical window of opportunity for the prevention and management of maternal and newborn complications, which can otherwise prove fatal. A large “proportion of” newborn illnesses and deaths can also be prevented using simple, low-cost interventions during delivery and the week following partum [9]. Reducing neonatal mortality is increasingly important not only because deaths that occur during the neonatal period is increasing as under-five mortality declines but also health interventions needed to address the major causes of neonatal deaths generally differ from those needed to address other under-five deaths [10]. A significant proportion of these neonatal deaths could be prevented by the appropriate management of the neonate presenting complications, such as very low birth weight, < 30 gestational weeks at birth or an Apgar score at the 5th minute of life < 7 [11].

Analysis of different studies in Ethiopia showed that the incidence of the neonatal mortality rate was ranged from 17.2 to 35.5 per 1000 live births [12–17]. The most determinate factors which were identified by previous studies were birth order, frequency of antenatal care, delivery place, twin delivery and size of neonate [12, 18, 19]. Birth asphyxia, neonatal infections, and prematurity were the three leading causes of neonatal mortality [12, 13].

Newborns in Ethiopia gaining attention through the Global Maternal Child Survival Program: Contributes to reductions of neonatal morbidity and mortality through capacity-building in high-impact services both at the community and the primary health care unit levels. The activity supports the government of Ethiopia to improve community maternal and newborn health practices and care-seeking behaviors; increases the provision of quality community-based newborn care services including management of newborn sepsis, and strengthens the supportive systems with a focus of district capacity building [20]. This program is underway, but to scale up a comprehensive way of implementation identifying determinate factors intensively is very important to reduce neonatal mortality further. Different studies were conducted in assessing determinates for neonatal mortality but there is a need to assess the immediate postnatal (within 2 days following delivery) cause of neonatal mortality that the majority of deaths occurred at that time. Therefore, there is a need for research in public hospitals of Gamo and Gofa Zones to assess the incidence, underlying causes and determinate factors for neonatal mortality.

## Methods

### Study setting, period and design

This prospective follow up study was conducted in public hospitals of Gamo and Gofa Zones from April 5, 2018, to March 5, 2019. There are six hospitals in Gamo and Gofa Zones but this study was done in selected three public hospitals (Arba Minch General Hospital (AMGH), Sawla General Hospital (SGH) and Chencha Primary Hospital (CPH)). The total population of the study area is 2,019,687. The estimated number of women of reproductive age (15–49) is 470,587 from this, the estimated number of delivery is 69,881 and the estimated number of live birth is 69,881. In Gamo and Gofa Zone, the institutional skilled delivery rate is 51.2% [21, 22].

### Sample size determination

Epi info7 software Stat Cal was used to estimate the sample size for this study. The assumptions used were prevalence of neonatal mortality among unexposed group (gestational age greater than 37 weeks) was 2.9% (p1 = 0.029) and the prevalence of neonatal mortality among exposed group (gestational age less than 37 weeks) was 5.8 (p2 = 0.058) from the study conducted in Southwest Ethiopia [12], 95% level of confidence, power of 90, and the ratio of 1:1. So, the calculated sample for this study was 2433 after adding a non-response rate of 10%. But, the sample size used for this study was 6769 based on the number of live births in the respective hospitals in 1 year period.

### Data collection tool

A structured interviewer-administered pre-tested questionnaire and standard abstraction checklist to review data from medical records were used to collect the data. The tools were developed adapted by reviewing different works of literature. A standard verbal autopsy (VA) questionnaire was used to collect the data on causes of neonatal death. The tool was developed and validated by WHO, Johns Hopkins University (JHU) and the London School of Hygiene and Tropical Medicine [23] (Additional files 1 and 2).

### Data collection procedures

A well-trained six BSc holder midwives prospectively identified neonates who experienced mortality cases during the follow-up period. As this was a prospective follow-up study; data were collected in different phases: In the first phase: all the baseline information in the hospital was collected either by interviewing or by abstracted from medical records. The data were collected from the delivery ward, postnatal ward and neonatal intensive care unit (NICU) of each hospital. For the neonates that died in the hospital stay, VA was conducted at a point in time, and case notes were used to collect the information. But, for those neonates who survived in the hospital stay the second phase proceeded at the end of the neonatal period. So, newborns were assessed for mortality cases whether they died within 28 days of life or survived and for those who don’t survive VA was conducted.

### Study variables

Neonatal mortality case was the dependent variable and socio-demographic and economic characteristics, maternal factors, maternal and child health services and obstetric factors were independent variables for this study.

### Data quality control

To ensure quality, the questionnaire was initially drafted in the English language and then translated into the local language, *“Gammogna and Amharic”* by verified translators. Finally, before data collection again re-translated back to English. The questionnaires were pre-tested in another hospital with a similar status to maintain the reliability and standard VA tool and the abstraction checklist was used to ensure the validity. Besides, extensive training was given for data collectors and supervisors. Data were checked for completeness, accuracy, clarity, and consistency before data entry into the software. Proper coding and categorization of data were maintained for the quality of the data to be analyzed. Double data entry was done for its validity and compare to the original data.

### Data analysis and processing

Data were coded, cleaned, edited and entered into Epi data version 3.1 and then exported to Stata version 15 for analysis. Binary logistic regression was done to see the association between each independent variable and outcome variable. A log-likelihood ratio (LR) was tested for the goodness of fit. All variables with *P* < 0.25 in the bivariate analysis were included in the final model of multivariable analysis to control all possible confounders. Variance inflation factor (VIF) > 10 and Tolerance (T) < 0.1 were considered as suggestive of the existence of multi co-linearity. A crude and adjusted Beta (β) coefficient with 95%CI was estimated to identify determinates for the neonatal mortality cases. In this study *P*-value < 0.05 was considered to declare a result as a statistically significant association.

## Results

### The overall process of the study

**Fig. 1:**
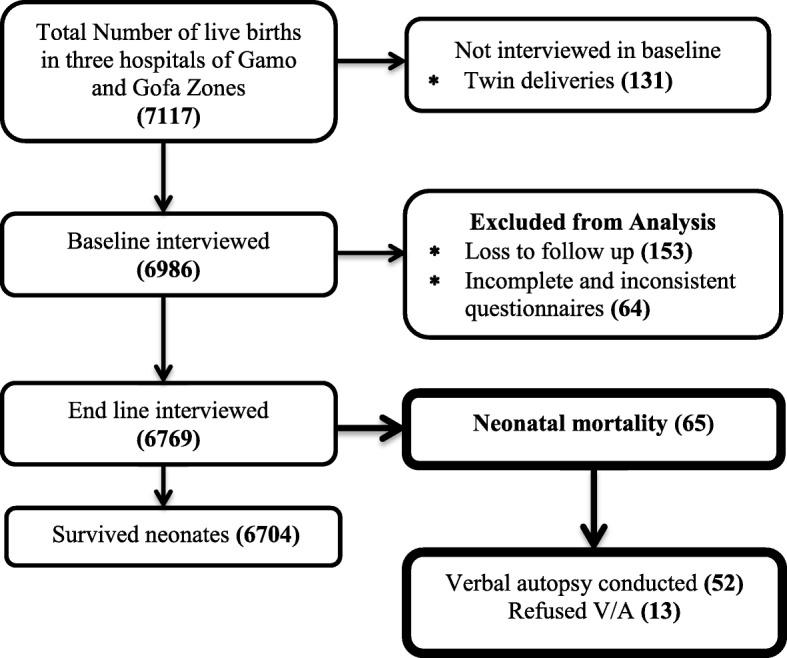
Overall process of the study conducted in public hospitals of Gamo and Gofa Zones, Southern Ethiopia, 2018/9

In this study, 6986 study participants were interviewed in the baseline after excluding 131 twin deliveries from total live births in three selected public hospitals from two zones of Southern Ethiopia. During follow up for 28 days 153 study participants became lost to follow up and 64 were excluded from the study because of inconsistent and incomplete information. At the end of follow up, 6769 study participants stayed in the cohort and interviewed the end line which gave a response rate of 96.9%. During follow up 6704 neonates were survived and 65 died. A verbal autopsy had conducted among 52 died neonates and the rest were refused (Fig. 1).

### Socio-demographic and economic characteristics of the respondents

Of the neonate’s mother, 3384 (50.0%) were age ranged 25–34 years old and with mean and standard deviation (SD) of 25.3 ± 5.02. The majority (95.0%) were married and 3727 (55.0%) had from the Gamo ethnicity group. Regarding the educational status of neonates mother, 1439 (21.3) had no formal education and 2069 (30.6%), 1822 (26.9%) and 1439 (21.3%) had primary (grade 1–8), secondary (grade 9–12) and college and above respectively. Two thousand eighty (30.7%) of the neonate’s father had the educational status of college and above and 2497 (36.9%) had merchant. Out of neonates mother 2885 (42.6%) had Orthodox religion follower and 420 (6.2%), 2966 (43.8%), 451 (6.7%) and 47 (0.7%) were Catholic, Protestant, Muslim and traditional respectively. More than half (57.7%) of the neonates mother was housewife and 1057 (15.6%), 1403 (20.7%), 123 (1.8%) and 283 (4.2) were merchant, government employer, daily labor and student respectively and 4067 (60.1%) had urban residents (Table 1).

**Table 1:** Socio-demographic and economic characteristics of study participants in public hospitals of Gamo and Gofa Zones, Southern Ethiopia, 2018/9 (*n = 6769*)

Characteristics	Frequency	Percentage
Age		
15–24	3002	44.3
25–34	3384	50.0
≥ 35	383	5.7
Marital status		
Married	6430	95.0
Other^a^	339	5.0
Ethnicity		
Gamo	3725	55.0
Gofa	1519	22.4
Other^b^	1525	22.6
Educational status of the father		
No formal education	1026	15.2
Primary (1–8)	1636	24.2
Secondary (9–12)	2027	29.9
College and above	2080	30.7
Occupation of the father		
Farmer	1773	26.2
Merchant	2497	36.9
Government employer	1974	29.2
Wavier	275	4.1
Daily laborer	250	3.7
The average income per month		
< 70.8USD	1775	26.2
70.8-177USD	3195	47.2
> 177USD	1799	26.6

^a^single, divorced and separated due to work

^b^Zayise, Amhara, Oromo, Gurage, Woliata, Konso, Derashe, Oyida, and Gidicho.

### Maternal and child health, and obstetric factors

Out of the neonate’s mother, 3900 (57.6%) had multipara (birth order ≥2), only 350 (9.0%) had a history of the stillbirth and 434 (11.1%) encountered loss of conceptus. Two thousand eight hundred (71.8%) of the mothers of the neonates were birth inter of 24–48 month and 329 (8.4%) had a history of neonatal death. Of the neonate’s mothers, 6004 (88.7%) had antenatal care (ANC) and 6674 (98.6%) had immediate postnatal care. Regarding mode of delivery 4943 (73.0%) gave birth by spontaneous vaginal delivery, 243 (3.6%) were instrumental and 1583 (23.4) were by caesarean section. One thousand two hundred sixty-two (18.6%) encountered prelabour rupture of membrane and 524 (7.7%) developed hypertension (HTN) during pregnancy. Out of the neonate’s mothers, 193 (2.9%) had anemic and 682 (10.1%) faced dystocia. From those who faced labor dystocia, 24 (3.5%) had due to uterine pre-rupture, 465 (68.2%) had due to prolonged labor and 193 (28.3%) had due to feto-pelvic disproportion. Two hundred thirty (3.4%) of the neonate’s mothers encountered infection and 130(1.9%) had developed other pathologies. Of the mothers who developed infection 33 (14.4%) had an unspecified infection and 50 (21.7%), 100 (43.5%) and 47 (20.4%) had puerperal endometritis, pyelonephritis and others (syphilis and malaria) respectively. From the mothers who developed other pathologies 33 (25.4%) had HIV/AIDS, 58 (44.6%) had heart diseases and 39 (0.3%) had others (DM, thyroid disorder, embolism, and DIC). Regarding the presentation of neonates, 5818 (86.0%) delivered with vertex and 3606 (53.3%) were male neonates. Of the neonates, 65 (1.0%) encountered birth trauma during delivery. From those 24 (36.9%) of the neonates had cephalhematoma, 9 (13.8%) developed caput succedaneum and 32 (49.3%) had others (fracture, bruising and subgleal hemorrhage) (Table 2).

**Table 2:** Maternal and child health and obstetric factors of study participants in public hospitals of Gamo and Gofa Zones, Southern Ethiopia, 2018/9 (*n = 6769*)

Variables	Frequency	Percentage
Number of ANC visit		
No visit	765	11.3
1–3	1820	26.9
≥ 4	4184	61.8
Hemorrhage		
Yes	315	4.7
No	6454	95.3
Cause of hemorrhage		
Placenta praevia	108	34.3
PPH	153	48.6
Other^a^	54	17.1
Prelabour rupture of membrane		
Yes	1262	18.6
No	5507	81.4
Hypertension during pregnancy		
Yes	524	7.7
No	6245	92.3
Classification of HTN		
Pre-eclampsia	297	56.7
Eclampsia	74	14.1
Chronic hypertension	77	14.7
Gestational hypertension	76	14.5
Presentation		
Vertex	5818	86.0
Non-vertex^b^	951	14.0
Sex of the neonates		
Male	3606	53.3
Female	3163	46.7
Gestational age		
< 37 week	808	11.9
≥ 37 week	5961	88.1
Birth weight		
< 2500 g	600	8.9
≥ 2500 g	6169	91.1
Baby referred to other health facilities		
Yes	77	1.1
No	6692	98.9

^a^accreta/increta/percreta, hemorrhage during delivery, uterine rupture, and other obstetric hemorrhages, and ^b^breech, transverse, face, and brow.

### Incidence of neonatal mortality

In this study inter and intra-hospital neonatal mortality incidence ratio was estimated with a 95% level of confidence per 1000 live births. The highest proportion of neonatal mortality was reported from Chencha Primary Hospital that 1.0% (95%CI: 0.5, 2.20%) Overall, neonatal mortality incidence ratio in selected three public hospitals was 0.96% (95%CI: 0.75, 1.22%) (Table 3).

**Table 3:** Incidence of neonatal mortality among study participants in selected hospitals of Gamo and Gofa Zones, Southern Ethiopia, 2018/9 (*n = 6769*)

Name Hospital	n(%) of NM	Total number of live births	NMIR^a^
			with 95%CI per 1000 live births
AMGH	42 (64.6)	4455 (65.8)	9.4 (6.9,12.7)
CPH	8 (12.3)	794 (11.7)	10.1 (5.0,20.0)
SGH	15 (23.1)	1520 (22.5)	9.9 (5.9,16.3)
Overall	65 (100)	6769 (100)	9.6 (7.5,12.2)

^a^Neonatal mortality incidence ratio.

### Causes and timing of neonatal mortality

**Fig. 2:**
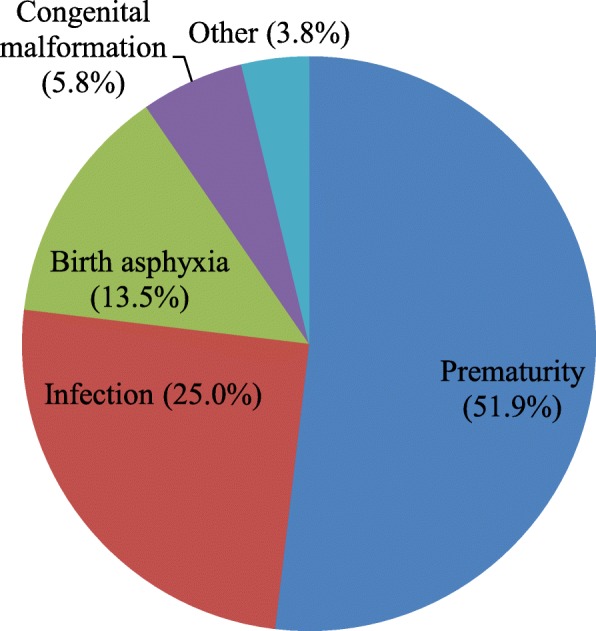
Causes of neonatal mortality among study participants in public hospitals of Gamo and Gofa Zones, Southern Ethiopia, 2018/9 (*n = 6769*)

**Fig. 3:**
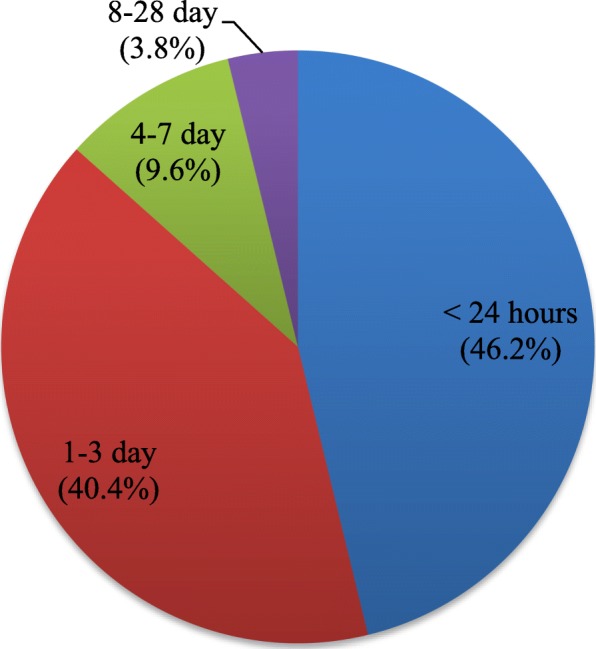
Timing of neonatal mortality among study participants in public hospitals of Gamo and Gofa Zones, Southern Ethiopia, 2018/9 (*n = 6769*)

In this study, 65 neonatal deaths occurred during the follow-up period in selected three public hospitals of Gamo and Gofa Zones, Southern Ethiopia. Of the neonatal deaths, only 52 respondents were agreed and interviewed for verbal autopsies but rest were refused for verbal autopsy. Almost half (51.9%) of the neonatal deaths were happened due to prematurity or gestational age less than 37 week, 13 (25%) due to neonatal infection, 7 (13.5%) were by birth asphyxia, 3(5.8%) congenital malformation due congenital malformation and the rest were with unspecified cause (Fig. 2). Regarding the timing of neonatal deaths, 24 (46.2%) died within 24 h, and 2 (3.8%) died after the day 8 up to 28 days (Fig. 3).

### Determinates of neonatal mortality

After adjusting in the multivariable model age of the mother, the number of ANC visits, presentation, gestational age at birth, and sex of the neonate were significantly associated with neonatal mortality. Advanced maternal age above 35 years old increased neonatal mortality significantly as compared to the age group 15 to 24 years old (β =1.34; 95% CI:0.54,2.14). The antenatal care visit of four or more significantly reduced neonatal mortality as compared to those who have no visit (β = − 0.88; 95% CI:-1.54,-0.21). Non-vertex presentation (β =1.15; 95% CI: 0.59, 1.69), gestational age of less than 37 week (β =1.18; 95% CI: 0.46, 1.89), and being male neonate (β =0.91; 95% CI: 0.21, 1.61) had significantly increased neonatal mortality (Table 4).

**Table 4:** Bivariate and multivariable analysis of determinates for neonatal mortality among study participants in selected hospitals of Gamo and Gofa Zones, Southern Ethiopia, 2018/9 (*n = 6769*)

Variables	Crude estimate β	Adjusted estimate β
	95%CI	
Place of residence		
Urban	−0.69(−1.19,-0.20)	− 0.29(− 0.87,0.27)
Age of the mother		
25–34	− 0.26(− 0.86,0.34)	−0.71(− 1.48,0.06)
≥ 35	2.07 (1.47,2.66)	1.34 (0.54,2.14)*
Birth interval		
Not applicable (primi)	NA	NA
< 24 month	−1.34(−2.74,0.06)	−0.65(− 2.27,0.98)
24–48 month	− 0.66(− 1.86,0.53)	−0.38(− 1.76,0.99)
Number of ANC visits		
1–3 visit	−1.77(− 2.42,-1.12)	−0.64(− 1.36,0.08)
≥ 4 visit	−2.12(− 2.68,-1.57)	− 0.88(− 1.54,-0.21)*
Party		
Multipara	0.37(− 0.15,0.88)	0.49(− 1.05,2.03)
Prelabour rupture of membrane		
Yes	1.08 (0.58,1.58)	0.34(−0.31,0.99)
Presentation		
Non-vertex^a^	1.56 (1.06,2.05)	1.15 (0.59,1.69)*
Gestational age		
< 37 week	1.94 (1.45,2.43)	1.18 (0.46,1.89)*
Birth weight		
< 2500 g	2.22 (1.73,2.72)	0.66(−0.07,1.39)
Sex of the neonate		
Male	1.47 (0.82,2.12)	0.91 (0.21,1.61)*

^a^breech, transverse, face and brow, NA: not applicable and *Significant at *P* < 0.05.

## Discussion

In this study neonatal mortality incidence ratio were 9.6(95%CI: 7.5, 12.2) per 1000 live births. Age of the mother, number of ANC visit, non-vertex presentation, gestational age, and sex of the neonate had significant risk factor for neonatal mortality. The major causes of neonatal mortality were prematurity, infection, and birth asphyxia.

The incidence of neonatal mortality was lower than studies done in northern Ethiopia (18.6 per 1000 live births), Kersa Health and Demographic Surveillance system site in Ethiopia (27.5 per 1000 live births) and two studies in southwest Ethiopia (35.5 and 27 per 1000 live births). But, it was higher than one study done in South Central Ethiopia (4.8 per 1000 live births) [12, 14–17]. The reason for this is the study period difference along with advance in the health care system that people’s attitudes and awareness about conditions that put the newborn for ill health and increase in health-seeking behavior from time to time. The causes of neonatal mortality (prematurity, infection, birth asphyxia, and anomalies) in this study were in line with different studies done in Ethiopia [12, 15, 16, 19].

Advanced maternal age (age greater than or equal to 35 years old) had a significant risk factor for neonatal mortality as identified in this study. This is the fact that advanced maternal age increases the risk that predisposes for different complications for the fetus, and for the neonates as well as for the mother. As indicated in this study, the non-vertex presentation was a determinate factor for neonatal mortality. This was in line with studies done in Southeast Brazil, South Africa, Uganda, and two studies in Ethiopia [12, 13, 16, 24, 25]. The reason for this is that non-vertex presentation is one of the major contributors for prolonged as well as obstructed labour which predisposes the neonate for life-threatening conditions and even for loss of life during the neonatal period.

A number of the ANC follow up had significantly reduced the risk of neonatal mortality as point out in this study. This is congruent with studies done in Southeast Brazil, and three studies in Ethiopia [12, 16, 19, 24]. This is obvious that the pregnant mother avoids preventable risk factors after having several ANC follow up, early identification and treatment of pre-existing conditions, and early screening of conditions that occur during pregnancy. In this study, gestational age less than 37 weeks was the determinate factor for neonatal mortality. This was consistent with the study done in Ethiopia [12]. This is because those newborns whose gestation age less than 37 weeks (preterm) are more likely to develop different complications during and after delivery and results for severe morbidity and mortality.

Being a male neonate was a significant risk for neonatal mortality as showed in this study. This is in line with some of the studies done in Ethiopia [16, 18, 19]. This is maybe due to the nature that male neonates more risk for different complication as stated in many studies.

The public health importance of this study is: Neonates are the risk population group for different complications and most likely affected by preventable causes of morbidity and mortality. Nowadays the neonatal mortality is on the way of decreasing but it is not that much satisfactory as compared to under-five child mortality. So, studies on risk factors that predispose the newborn for ill health and mortality are very important to prevent the underlining causes and to give immediate solutions.

The main strength of this study that the design was prospective follow up that it gave a true measure of the incidence of neonatal mortality and to develop cause and effect relationship. Standard and validated verbal autopsy tool was used to measure the causes for neonatal mortality to maintain the validity and reliability. The large sample size was used for this study that resulted in high power and greater precision.

The limitations are: response of the verbal autopsy was written based on the respondent’s view and some of the causes were difficult to classify in one category. Besides, during follow up some mothers did not come to health care institutions for immunization as well as for other services and very challenged to trace those mothers as they were out of health facilities.

## Conclusions

This study showed that the incidence of neonatal mortality ratio was optimum. Age of the mother, number of ANC visits, non-vertex presentation, gestational age, and sex of the neonate had significant risk factor neonatal mortality. The major causes of neonatal mortality were prematurity, infection, and birth asphyxia as identified by this study. Both maternal and neonatal risk factors for neonatal mortality were identified in this study. Therefore, early identification of obstetric complications and immediate interventions, strengthen antenatal care services both at the community as well as in the health care institutions, screening the conditions early during intrapartum and postnatal period to give immediate measures to avoid preventable causes of neonatal mortality. The health professionals are responsible to provide quality antenatal care services for pregnant mothers both at health care institutions and in the community. The community is also responsible to seek health information during the prenatal and postnatal period which is provided by health professionals and put in practice. Other scholars should incorporate some of the variables that are not addressed in this study such as wealth index, nutritional status, and cultural aspects. It is also very important if the mixed study design is applied.

## Supplementary information

**Additional file 1.** Tool

**Additional file 2.** STROBE checklist

## Data Availability

The data will not be shared to preserve participant anonymity.

## Abbreviations

ANC: Antenatal Care
HTN: Hypertension
NICU: Neonatal intensive care unit
NM: Neonatal mortality
VA: Verbal Autopsy

## References

[1] Sustainable Development Goal 3: Ensure healthy lives and promote well-being for all at all ages. Progress of goal 3 in 2017. Reproductive, maternal, newborn and child health. Available at: https://www.who.int/sdg/targets/en/. Accessed 27 Dec 2018.

[2] WHO, Sustainable Development Goal 3 (2017). Health. SDG 3 “ensure healthy lives and promote wellbeing for all at all ages”.

[3] Kamath-Rayne BD, Thukral A, Visick MK (2018). Helping babies breathe, second edition: a model for strengthening educational programs to increase global newborn survival. Glob Health Sci Pract.

[4] Ethiopian Public Health Institute (EPHI) [Ethiopia] and ICF (2019). Ethiopia mini demographic and health survey 2019: key indicators.

[5] WHO (2018). Global Health Observatory (GHO) data. Neonatal mortality, world health statistics data visualizations dashboard.

[6] Central Statistical Agency (CSA) [Ethiopia] and ICF (2016). Ethiopia demographic and health survey 2016: key indicators report.

[7] World Health Organization (WHO) (2013). Counseling for maternal and newborn health care: a handbook for building skills.

[8] WHO Reproductive Health Library (2018). WHO recommendation on postnatal discharge following uncomplicated vaginal birth. The WHO reproductive health library.

[9] UNICEF (2016). Maternal and newborn health.

[10] WHO. Global Health Observatory (GHO) data (2016). Neonatal mortality situation, and trends.

[11] Pileggi C, Souza JP, Cecatti JG, Faundes A. Neonatal near-miss approach in 2005 WHO Global Survey Brazil. J Pediatr (Rio J). 86(1):21–6. 10.2223/JPED.1965 20151094

[12] Debelew Gurmesa Tura, Afework Mesganaw Fantahun, Yalew Alemayehu Worku (2014). Determinants and Causes of Neonatal Mortality in Jimma Zone, Southwest Ethiopia: A Multilevel Analysis of Prospective Follow Up Study. PLoS ONE.

[13] Nakimuli A, Mbalinda SN, Nabirye RC, Kakaire O, Nakubulwa S, Osinde MO, Kakande N, Kaye DK. Stillbirths, neonatal deaths and neonatal near-misscases attributable to severe obstetric complications: a prospective cohort study in two referral hospitals in Uganda. BMC Pediatr. 15: p. 44. 10.1186/s12887-015-0362-3 PMC4416266 25928880

[14] Yirgu Robel, Molla Mitike, Sibley Lynn (2017). Determinants of neonatal mortality in rural Northern Ethiopia: A population based nested case control study. PLOS ONE.

[15] Assefa N, Lakew Y, Belay B, Kedir H, Zelalem D, Baraki N, Damena M, Oljira L, Ashenafi W, Dedefo M. Neonatal mortality and causes of death in Kersa Health and Demographic Surveillance System (Kersa HDSS), Ethiopia, 2008-2013. Maternal Health Neonatol Perinatol. 2:7. 10.1186/s40748-016-0035-8 PMC4950756 27437118

[16] Gizaw M, Molla M, Mekonnen W. Trends and risk factors for neonatal mortality in Butajira District, south Central Ethiopia, (1987–2008): a prospective cohort study. BMC Pregnancy Childbirth. 14:64. 10.1186/1471-2393-14-64 PMC3923005 24517305

[17] Yaya Yaliso, Eide Kristiane Tislevoll, Norheim Ole Frithjof, Lindtjørn Bernt (2014). Maternal and Neonatal Mortality in South-West Ethiopia: Estimates and Socio-Economic Inequality. PLoS ONE.

[18] Mekonnen Y, Tensou B, Telake DS, Degefie T, Bekele A. Neonatal mortality in Ethiopia: trends and determinants. BMC Public Health. 13:483. 10.1186/1471-2458-13-483 PMC3659057 23683315

[19] Kolola Tufa, Ekubay Meseret, Tesfa Endalamaw, Morka Wogene (2016). Determinants of Neonatal Mortality in North Shoa Zone, Amhara Regional State, Ethiopia. PLOS ONE.

[20] Kale PL, Mello-Jorge MHP, Silva KSD, Fonseca SC. Neonatal near miss and mortality: factors associated with life-threatening conditions in newborns at six public maternity hospitals in Southeast Brazil. Cad Saude Publica. 33(4):e00179115. 10.1590/0102-311X00179115 28538795

[21] Federal Democratic Republic of Ethiopia Central Statistical Agency. Population Projection of Ethiopia for All Regions: At Wereda Level from 2014 – 2017. Addis Ababa, Ethiopia; 2013. Available at: file:///C:/Users/user/Downloads/Population%20projection%20of%20Ethiopia%20for%20all%20Regions%20at%20wereda%20level%20from%202014%20-%202017.pdf. Accessed 12 Jan 2018.

[22] Gamo Gofa Zone health bearue department report, 2007 EC.

[23] Anker M, Black RE, Coldham C (1999). A standard verbal autopsy method for investigating causes of death in infants and children.

[24] Kale PL, et al. Neonatal near miss and mortality: factors associated with life-threatening conditions in newborns at six public maternity hospitals in Southeast Brazil. Cad Saude Publica. 33(4):e00179115. 10.1590/0102-311X00179115 28538795

[25] Avenant T (2009). Neonatal near miss: a measure of the quality of obstetric care. Best Pract Res Clin Obstet Gynaecol.

