# Supplementary material for: Neonatal mortality and its determinates in public hospitals of Gamo and Gofa zones, southern Ethiopia: prospective follow up study
Source: BMC Pediatr. 2019 Dec 16;19:499. doi: 10.1186/s12887-019-1881-0 (PMC6912940; doi:10.1186/s12887-019-1881-0)
Supplement: Supplementary file 1 — Additional file 1. Tool [file 12887_2019_1881_MOESM1_ESM.pdf]

English Version Tool

Phase one Interview and Record Review ***Identification Number***

SNo	Questions	Response	Skip
**Part I: Identifications**			
101	Hospital Name	a. AMGH<br>b. Chencha Primary Hospital<br>c. Sawla General Hospital	
102	Hospital code	____/___/____	
103	Date form filled to start	____/___/____	
104	Woreda/District	__________________________	
105	Kebele	__________________________	
106	Village	__________________________	
107	Phone number (anyone who are immediate response)		
108	Health Center in the surround		
109	Health Post in the surround		
110	Health extension worker name and phone number		
111	Admission date	_____/____/____EC	
112	Maternity record number/Medical Reg. Number	__________________	
113	Admission mode	a. Self-referred<br>b. Referred from another facility	
114	Means of transport used	a. Ambulance<br>b. Public transport<br>c. Personal vehicle<br>d. Other, specify____________	
**Part II: Socio-demographic and economic characteristics**			
201	How old are you?	____________(in completed year)	
202	What is your marital status?	a. Married<br>b. Single<br>c. Widowed<br>d. Divorced<br>e. Separated due to work	
203	What is your ethnicity?	_______________________	
204	What is mother's educational status?	a. No formal education<br>b. Primary(1-8)<br>c. Secondary(9-12)<br>d. College and above	
205	What is paternal educational status?	a. No formal education<br>b. Primary (1-8)	

		c. Secondary (9-12)<br>d. College and above	
206	What is your religion?	a. Orthodox<br>b. Catholic<br>c. Protestant<br>d. Muslim<br>e. Traditional	
207	What is the occupation of the mother?	a. House wife<br>b. Merchant<br>c. Government employer<br>d. Daily laborer	
208	What is the occupation of the father?	a. Farmer<br>b. Merchant<br>c. Government employer<br>d. Wavier<br>e. Daily laborer	
209	Where is the place of residence?	a. Urban<br>b. Rural	
210	How much is your family average income per month?	______________(ETB)	
**Part III: Maternal Factors related questions (Record review if it is available, if not interview the mother)**			
301	Number of pregnancies (gravidity)?	___________(in number)**If primi** →	**401**
302	Number of birth orders (parity)?	___________ (in number)	
303	Do you pervious history of still birth?	a. Yes<br>b. No	
304	Do you pervious history of abortion?	a. Yes<br>b. No	
305	The duration between the current birth and the preceding birth in months?	___________ (in months)	
306	Do you have pervious history of neonatal death?	a. Yes<br>b. No	
**Part IV: Maternal and child health care questions (similar with part III)**			
401	Did the woman receive antenatal care?	a. Yes<br>b. No →	**403**
402	If yes, number of visits	________________	
403	Do you have immediate PNC visit?	a. Yes<br>b. No	
**Part V: Obstetric related questions (Record review/interview)**			
501	Date of delivery or end of pregnancy	____/___/____	
502	Mode of delivery	a. Spontaneous vaginal delivery<br>b. Instrumental assisted delivery<br>c. Caesarean section	

503	Hemorrhage	a. Yes<br>b. No →	505
504	If, yes	a. Placenta praevia<br>b. Placenta accreta/increta/percreta<br>c. Retro-placental haematoma<br>d. Other first trimester hemorrhage<br>e. Hemorrhage during delivery (no other specification)<br>f. Uterine rupture<br>g. Postpartum hemorrhage (no other specification)<br>h. Other obstetric hemorrhage	
505	Premature rupture of member	a. Yes<br>b. No	
506	Hypertension	a. Yes<br>b. No →	508
507	If, Yes	a. Pre-eclampsia<br>b. Eclampsia<br>c. HELLP<br>d. Chronic hypertension<br>e. Gestational hypertension	
508	Anaemia (Hb < 11g/dl)	a. Yes<br>b. No →	510
509	If, Yes		
	a. Haemoglobin level (g/dl)	____________	
	b. Unknown		
510	Infection	a. Yes<br>b. No →	512
511	If, Yes	a. Unspecified infection<br>b. Puerperal endometritis<br>c. Pyelonephritis<br>d. Septicaemia<br>e. Peritonitis<br>f. Parietal suppuration<br>g. Malaria<br>h. Syphilis<br>i. Other systemic infection	
512	Dystocia	a. Yes<br>b. No →	514
513	If, Yes	a. Uterine pre-rupture<br>b. Prolonged labour<br>c. Foeto-pelvic disproportion	
514	Other pathologies	a. Yes<br>b. No →	516

515	If, Yes	a. HIV/AIDS<br>b. Embolic diseases (thrombosis/amniotic fluid or gaseous embolism<br>c. Heart disease<br>d. Sickle-cell disease<br>e. Other (specify: ___________)	
516	**Newborn**		
	**A.** Presentation	a. Chephalic<br>b. Breech<br>c. Transverse/face/brow<br>d. Other, specify___________	
	**B.** Sex	a. Male<br>b. Female<br>c. Unknown	
	**C.** Birth trauma	a. Yes<br>b. No	
	**D.** If the above response is yes	Specify, ________________	
	**E.** Baby referred to another facility?	a. Yes<br>b. No	
	**F.** Admitted to special care or intensive care unit?	a. Yes<br>b. No	

Fill This Check List If the Neonate Died

The respondent for this check list will be either health care provider or anyone who are relationship with deceased neonate

**Part VII: Check List Two (only fill if the neonate is died)**			
601	**Does the newborn died after developing near miss criteria?**	**Yes**<br>**No** →	 **Stop**

Informed Consent Statement

Hello. My name is ____ and I am working with Arba Minch University. We are collecting information on the causes of death. We would very much appreciate your participation in this effort. We want to ask you about the circumstances leading to the death of the deceased. Whatever information you provide will be kept strictly confidential. No information identifying you or the deceased will ever be released to anyone outside of this information-collection activity.

Participation in this survey is voluntary and you can choose not to answer any individual question or all of the questions. You may also stop the interview completely at any time without any consequences at all. However, we hope that you will participate in this survey since the results will help the government improve services for people.

At this time, do you want to ask me anything about the purpose or content of this interview?

May I begin the interview now?

Signature of interviewer:____________________________ Date:________________

Respondent Agrees To Be Interviewed **CONTINUE,** Not Agree To Be Interviewed **STOP**

**Causes of Neonatal death**			
602	Is the mother alive at the time of interview?	a. Yes<br>b. No	
603	Is the main respondent is health care provider?	a. Yes →<br>b. No	**605**
604	If No, What is the relationship of the main respondent to deceased neonate?	a. Mother<br>b. Father<br>c. Grandfather<br>d. Grandmother<br>e. Uncle<br>f. Aunt<br>g. Other (Specify) _____	
605	Where was the neonate died?	a. Hospital<br>b. Health center<br>c. Health post<br>d. On route to health facility<br>e. Home<br>f. Other (specify) ______	
606	If the death was at health facility, record the facility name and address.	Facility name__________<br>Date of admission ______	

Open history question

607. Could you please tell me about the illness that led to the death of the neonate?
Instruction to interviewer: allow the respondent to tell you about the illness that lead to the death of the neonate in her/his own words. Do not prompt except for asking "was there anything else" until the respondent says there was nothing else.

____
____
____
____
____
____
____
____
____
____

608. Take a moment to circle all items mentioned spontaneously in the open history questionnaire. Use the following to guide you through the rest of the questionnaire.
1. Diarrhea 2. Cough 3. Fever 4. Rash 5. Injure 6. Coma 7. Fit 8. Stiff neck 9. Tetanus
10. Measles 11. Kwashiorkor 12. Marasmus 13. Difficult breathing 14. Rapid breathing
15. Complicated delivery 16. Malformation 17. Very small at birth 18. Very thin 19. Born early
20. Pneumonia 21. Malaria 22. Jaundice 23. Other terms (specify)___________

609	What was the length of time the neonate was ill before he/she died?	______(days)	
610	Was care (treatment) sought outside home while the neonate had this illness?	a. Yes<br>b. No →	**612**
611	If Yes, from where was the care sought? (Multiple responses are possible, so prompt anywhere else)<br>1. Government hospital<br>2. Government health center<br>3. Government health post	a. Government hospital<br>b. Government health center<br>c. Government health post<br>d. Private clinic<br>e. Private pharmacy, drug store or drug vender<br>f. Other, specify_______	
612	Did the deceased neonate have any malformations at birth?	a. Yes<br>b. No →	**614**
613	If Yes, Where was the malformation? (Multiple answers are possible)	a. Head<br>b. Body<br>c. Arms/hands<br>d. Legs/feet<br>e. Other (Specify) _____	

614	Was the neonate able to breath after birth?	a. Yes<br>b. No	
615	Was the neonate able to suckle or bottle feed in a normal way after birth?	a. Yes<br>b. No	
616	Did the neonate stop suckling in a normal way after starting?	a. Yes<br>b. No →	**619**
617	How long before death was the neonate stop suckling?	a. Less than 1 day<br>b. 1-2 day<br>c. More than 2 days	
618	How long after birth (at what age) did the neonate stop suckling?	a. Less than 1 day<br>b. 1-2 day<br>c. 3-7 days<br>d. 8-14 days<br>e. 15-28 days	
619	Was the neonate able to cry after birth?	a. Yes<br>b. No	
620	Did the neonate stop being able to cry?	a. Yes<br>b. No →	**622**
621	If Yes, how long before death did the neonate stop suckling?	a. <1 day<br>b. 1 day or more	
622	During the illness that led to death, did the neonate have spasm or convulsions?	a. Yes<br>b. No	
623	During the illness that led to death, did the neonate have become unconscious?	a. Yes<br>b. No	
624	During the illness that led to death, did the neonate have bulging fontanel?	a. Yes<br>b. No	
625	During the illness that led to death, did the neonate have tetanus?	a. Yes<br>b. No	
626	During the illness that led to death, did the neonate have yellow discolouration of eyes?	a. Yes<br>b. No	
627	During the illness that led to death, did the neonate have redness or drainage from the umbilical cord stump?	a. Yes<br>b. No	
628	During the illness that led to death, did the neonate have areas of skin that were red and hot?	a. Yes<br>b. No	
629	During the illness that led to death, did the neonate have skin rash with bumps containing pus?	a. Yes<br>b. No	
630	During the illness that led to death, did the neonate have fever?	a. Yes, last for___days<br>b. No	
631	During the illness that led to death, did the neonate have frequent loose or liquid stools or diarrhea?	a. Yes, last for ____days<br>b. No	
632	During the illness that led to death, did the neonate cough?	a. Yes , last for ___ days<br>b. No	

633	During the illness that led to death, did the neonate have difficulty breathing?	a. Yes , last for ___ days<br>b. No	
634	During the illness that led to death, did the neonate have fast breathing?	a. Yes , last for ___ days<br>b. No	
635	During the illness that led to death, did the neonate ever stop breathing for long time and started again?	a. Yes<br>b. No	
636	During the illness that led to death, did the neonate have chest indrawing?	a. Yes<br>b. No	
637	During the illness that led to death, did the neonate have flaring nostrils?	a. Yes<br>b. No	
638	During the illness that led to death, did the neonate have pneumonia?	a. Yes<br>b. No	
639	Has the mother of the neonate ever been tested for HIV?	a. Yes<br>b. No →	**Stop**
640	If Yes, was the HIV test ever positive?	a. Yes<br>b. No	
641	Has the mother of the neonate ever been told that she had “AIDS” by health worker?	a. Yes<br>b. No	

Many Thanks!!!

Gammogna Version Questionnaire

Shempo1 Qalan oyishetizanne Karideppe Maxetiza Oyisha   Shakko payiddo

O.py	Oyisha	Zarro	Dhiko
**Basso 1: Shakko Oyishatta**			
101	Hospittale sunitha	a. Arba Mince ubba Hospittale<br>b. Cencha Koyiro Detha Hospittale<br>c. Sawila koyiro ubba Hospittleal	
102	Hospittale kode	____/___/____	
103	Oyishay oyisheto domidda gallasa	____/___/____	
104	Worrada	__________________________	
105	Kabale	__________________________	
106	Gutta	__________________________	
107	Silike payddo		
108	Guttan dizza xenna xabiya		
109	Guttan dizza xenna keela		
110	Xenna ekistenishine silike payiddo		
111	Hospittale gelida gallasa	_____/____/____	
112	Hospittale kardde payddo	__________________	
113	Hospittale yanas qofisiday	a. Taffe<br>b. Hara xenna go7a immiza keetha	
114	Ayibban yaddi	a. Amubulanissen<br>b. Dere gathiza makinani<br>c. Ta makinani<br>d. Harabba, yota____________	
**Basso II: Ayottaba Yottiza Hannota**			
201	Ayotta layitha	____________(kumetha layithan)	
202	Azzina gello hannota	a. Gelladus<br>b. Shagetadus<br>c. Azzinay hayqides<br>d. Gellabuku<br>e. Osso gishaw hara bittan dees	
203	Gita zare	_________________	
204	Ayyi timirite tamarida hannota	a. Timirte tamarabeyiku<br>b. 1-8 kifile<br>c. 9-12 kifile<br>d. Kollejenne kollejeppe adho	
205	Awwa timirite tamarida hannota	a. Timirte tamarabeyinna	

		b. 1-8 kifile<br>c. 9-12 kifile<br>d. Kollejenne kollejeppe adho	
206	Amanno	a. Orotodokisse<br>b. Katolike<br>c. Missonne<br>d. Issilamma<br>e. Balle ammano	
207	Ayyi osso	a. So ossanicha<br>b. Zali77e<br>c. Kawwo ossanicha<br>d. Wolikka osso	
208	Awwa osso	a. Gabarre<br>b. Zali77e<br>c. Kawwo ossanicha<br>d. Shemanne<br>e. Wolikka osso	
209	Dizza soho	a. Kattama<br>b. Gaxarre	
210	Aginani ayi kenna birra demmeti?	____________________Birra	
**Basso III: Ayyi hannota oyishata (Karideppe xella woykko ayyiyo oyisha)**			
301	Appunitho shaaradda?	__________(payiddon)**Koyirro** →	**401**
302	Appunitho yeladdi?	__________ (payiddon)	
303	Hayissape sinthan gawon hayiqiddi yeletidda yeloy dizze?	a. Dees<br>b. Baawa	
304	Hayissape sinthan shaara wodhoy dizze?	a. Dees<br>b. Baawa	
305	Ha shaaraninne koyrro shaaran ayyi kenna haakoy dizze?	__________ (agginani)	
306	Suutha na hayiqoy dizze?		
**Basso IV: Ayyotanne qerri nayitta hannota oyishata (Basso III maala kunitha)**			
401	Shaara kalethoy dizze?	a. Dees<br>b. Baawa →	**403**
402	Ayyi kenna kaaladi?	_______________	
403	Yeloppe guyye kaalethoy/innikibikaabey dizze?	a. Dees<br>b. Baawa	
**Basso V: Yelo hannottaba oyishata (karideppe xella woyikko oyisha)**			
501	Yelo gallasa?	____/___/____	
502	Ayibban yeladdi?	a. Medho oggen/maxenenni<br>b. Masaran madetada<br>d. Opereshinenni	
503	Suutha gogoy dizze?	a. Dees<br>b. Baawa →	**505**

504	Boolan dizza oyishas zaaroy dees gidiko	a. “Pilasenta priva”<br>b. “Pilasenta akirata/inkirata/perkirata”<br>c. “Retiro-pilacenta hematoma”<br>d. Harra koyirro hezantho aginna suutha googo<br>e. Yello bolla suutha googo (harra shaakoy baawa)<br>f. Yello keetha daketto<br>g. Yelloppe guye suutha googo (harra shaakoy)<br>h. Harra suutha googo	
505	Yelloppe sinthan uguntha googoy dizze?	a. Dees<br>b. Baawa	
506	Bolla giddon suuthan suugethay guujoy dizze?	a. Dees<br>b. Baawa →	**508**
507	Bolla dizza oyishas zaaroy dees gidiko,	a. “Pri-ikilapishiya”<br>b. “Ikilapishiya”<br>c. HELPPI<br>d. Gaamida suutha suugetha<br>e. Shaarethan bolla gaagiza suutha suugetha	
508	Suutha kuuthetez (Hb < 11g/dl)	a. Dees<br>b. Baawa →	**510**
509	Dees gidiko		
	a. Hemogilobinne (g/dl)	___________	
	b. Erettena		
510	“inifekishine” dizze?	a. Dees<br>b. Baawa →	**512**
511	Dees gidiko	a. Errotenna “inifekishine”<br>b. “Puriperal endometereyites”<br>c. Pyilonepirayites”<br>d. Suuttha marizetto<br>e. “Peritonayites”<br>f. “Parishal suupereshin”<br>g. Wooba’ugunno<br>h. “Sipilese”<br>i. Harra “sisetemike inifekishene”	
512	“Dayitoshiya” dizze?	a. Dees<br>b. Baawa →	**514**
513	Dees gidiki	a. Yello keetah daketo<br>b. Yello bolla gami77o<br>c. “Feto-pelivike disporoporishine”	
514	Harra “patojjine” dizze?	a. Dees	

		b. Baawa →	**516**
515	Dees gidiko?	a. HIV/AIDS<br>b. "Imibolike dizize (tirobosise/amiotikke woyikko gasheyes imibolizime"<br>c. Woozanna haarige<br>d. "Sikile-celle dizize"<br>e. Harra (yotta: __________)	
516	**Suutha na77a**		
	A. "Pirezentishine"	a. "Sepalike"<br>b. "Biriche"<br>c. "Tiransiverse/fase/birow"<br>d. Harra, yotta__________	
	B. "Xoota"	a. Adde<br>b. Maache<br>c. Erretenna	
	C. Yello bolla decheto	a. Dees<br>b. Baawa	
	D. Dees gidiko	yotta, _______________	
	E. Na77ay harra hospitale daaketidde?	a. Dees<br>b. Baawa	
	F. Na77ay "initensive kerre unite" geelide?	a. Geelides<br>b. Geelibenna	

Kaliddi dizza oyishata suutha na77ay hayiqida gidiko kuunitha

<table>
<tr><td colspan="4">Basso VII: Chekilisite 2 (Hayiqia na77as kunitha)</td></tr>
<tr><td>601</td><td>Na77ay hayiqide?</td><td>a. Hayiqides
b. Hayiqibena →</td><td>
Essa</td></tr>
<tr><td colspan="4">Piligethan Dossan Gelidayis Bessiza Basso
Hello. Suunithay____Ariba mince yuniversitten othayis. Suutha na77ita hayiqos gathida hannotata bolla maraja shishayis. Hayissa maraja shisho bolla gelidda gishas daroppe galatayis. Nuni shishida oyishay suutha na77ita hayiqos gathiza hannotata bolla. Neppe nuni shisha zaroy ubbay xuuren oyiketes. Neppene hayiqida suutha na77a hannotan shiqida zaroy oddesika immetena. Ha piligethan geeloy dosatethanne zaaro immanas koyota oyishetas zaaro immota aggoy danda7etes woyikko oyisha domidda guyedippe zarro immota aggoy danda7etes. Gidoppe attin neeppe eketiza zaaro suutha na77itas kooshiz xeena hannotas daroppe go77es.
Oyishay diiko oyichanas dandi77etes, oyishay dizze?
Oyisha doomo?
Oyisha oyishiza daana firima:____________________________ gaalasa:________________
Oyisha zaariza daanay erro giides <u>Dooma</u> oyisha zaariza daanay erro gibenna <u>Essa</u></td></tr>
<tr><td colspan="4">Hayiqos gaathiza hannotata</td></tr>
<tr><td>602</td><td>Hayiqidda na77as ayya de77iza?</td><td>a. Daasu
b. Duuku</td><td></td></tr>
<tr><td>603</td><td>Zaaro imizay xeena moya77icha?</td><td>a. Ee →
b. Deena</td><td>605</td></tr>
<tr><td>604</td><td>Zaaroy deena gidiko,</td><td>a. Ayyo
b. Awa
c. Awa awa
d. Awa ayyo
e. Ayi awa
f. Ayi ayyo
g. Harra (yotta) _____</td><td></td></tr>
<tr><td>605</td><td>Na77ay anani hayiqide?</td><td>a. Hosipitalen
b. Xeena xabiya
c. Xeena keela
d. Harra hakime keetha
e. Sohon
f. Harra (yotta) _____</td><td></td></tr>
<tr><td>606</td><td>Na77ay hayiqiday hakime keethan gidikko, haakime keetha sunitha, bootanne gallasa xaafa?</td><td>Sunitha_________
Gelidda gallasa ______</td><td></td></tr>
</table>

Paatethan zaaretiza oyisha

607. Na77ay waani hayiqidako yoota?

Oyisha oyishiza daana koyiro: zaaro zaariza daanay na77ay wanni hayiqidakko ba qaalan yotto. Zaaro zaariza daanas ogge immoppa "harraba diko attin" daanay oniganaw gaathaw.

____
____
____
____
____
____
____
____
____
____

608. Zaaro immiza daanay pilligethan zaarishin kaliddi de77eza hannotateppe kaababa..

1. Guuso 2. Quufe 3. Puusha/bolla maadunitha 4. Bolla qohetto 5. Baana baletto 6. Fiite 7. Qoppe qaxonitta aggo 8. Tetanese 9. Kuufuno 10. Kosharikore 11. Marasimese 12. Shempo bolla wayetho 13. Esson shemppo 14. Yello bolla woyetho 15. Bolla paacce 16. Kilo guutha na77a 17. Gaasha na77a 18. Agginay gaakonitta yeletto 19. Niimoniya 20. Wooba /ugunno 21. Bolla biicha giido 22. Haara ba (yootta)___________

609	Na77ay hayiqanappe sinthan ayyi keena saketidde?	______(gallasan)	
610	Hakime keetappe haara botan hakemetidde?	a. Hakemitides<br>c. Hakenitibena →	612
611	Boolan dizza oyisha zaaroy haakime keetha gidikko,	a. Hospitalen<br>b. Xeena xaabiyan<br>c. Xeena keelan<br>d. Giile haakime keethan<br>e. Giile parimaasen, dhaale keethan<br>f. Harraba (yoota)_______	
612	Na77ay bolla paacey dizze?	a. Dees<br>b. Baawa →	614
613	Bollan dizza oyishas zaaroy dees gidiko,	a. Huphe<br>b. Bolla<br>c. Kushe<br>d. Tooho<br>e. Haaraba (Yoota) _____	
614	Na77ay shemppe?	a. Shempes<br>b. Shemppene	
615	Na77ay dhami woykko xuuxo dhami?	a. Dhames<br>b. Dhamene	
616	Na77ay hayiqanappe sinthan dhamo woyikko shempo essire?	a. Essides<br>b. Essibenna →	619
617	Na77ay hayiqappe sinthan ayyi kennani dhamo essire?	a. 1 gallasappe sinthan<br>b. 1-2 gallasa<br>c. 2 gallasappe bollan	
618	Ayyi keena gallasan na77ay damo esseri?	a. 1 gallasappe shinthan<br>b. 1-2 gallasa<br>c. 3-7 gallasa<br>d. 8-14 gallasa<br>e. 15-28 gallasa	
619	Na77ay koyirro waasi?	a. Waases<br>b. Waasena	
620	Na77ay hayiqanappe sinthan waaso esseri?	a. Essires<br>b. Essibenna →	622
621	Bollan dizza oyishas zaaroy essires gidiko,	a. <1 gallasa<br>b. 1 gallasappe bolla	
622	Na77a hayiqos gathira saako saaketishin, esippazime/konivelizhiney dizze?	a. Dees<br>b. Baawa	
623	Na77a hayiqos gathira saako saaketishin, baana baaletidde?	a. Baaletides<br>b. Baaletibena	

624	Na77a hayiqos gathira saako saaketishin, huuphe phidhey puridde?	a. Purires<br>b. Puribenna	
625	Na77a hayiqos gathira saako saaketishin, did the neonate have tetansey dizze?	a. Dees<br>b. Baawa	
626	Na77a hayiqos gathira saako saaketishin, bolla bicatoy dizze?	a. Dees<br>b. Baawa	
627	Na77a hayiqos gathira saako saaketishin, gulla zo77otetha/ mizhigetay dizze?	a. Dees<br>b. Baawa	
628	Na77a hayiqos gathira saako saaketishin, bolla galibay zoo77ide/ miichey dizze?	a. Dees<br>b. Baawa	
629	Na77a hayiqos gathira saako saaketishin, bolla galiba shifita/ giishay dizze?	a. Dees<br>b. Baawa	
630	Na77a hayiqos gathira saako saaketishin, bolla michy dizze?	a. Dees, gamiday___gallasa<br>b. Baawa	
631	Na77a hayiqos gathira saako saaketishin, guusoy dizze?	a. Dees, gamiday___gallasa<br>b. Baawa	
632	Na77a hayiqos gathira saako saaketishin, quufoy dizze?	a. Dees, gamiday___gallasa<br>b. Baawa	
633	Na77a hayiqos gathira saako saaketishin, shemppo bolla wayithoy dizze?	a. Dees, gamiday___gallasa<br>b. Baawa	
634	Na77a hayiqos gathira saako saaketishin, esson shemppoy dizze?	a. Dees, gamiday___gallasa<br>b. Baawa	
635	Na77a hayiqos gathira saako saaketishin, shemppo essidappe guye doomoy dizze?	a. Dees<br>b. Baawa	
636	Na77a hayiqos gathira saako saaketishin, tiira siriphetoy dizze?	a. Dees<br>b. Baawa	
637	Na77a hayiqos gathira saako saaketishin, siidhe qaaxoy dizze?	a. Dees<br>b. Baawa	
638	Na77a hayiqos gathira saako saaketishin, goofinna miichay dizze?	a. Dees<br>b. Baawa	
639	Na77a ayya HIV maramaretadde?	a. Maramaretadus<br>b. Marameretabeyku →	**Essa**
640	Bollan dizza oyishas zaaroy maramaretadus gidiku wuxetey pozetive?	a. Eyye<br>b. Deena	
641	Hayiqdda na77a ayya "AIDS" dees gidi yootida xeena moyanichay dizze?	a. Dees<br>b. Deena	

Daroppe Gallatayis!!!

Amharic Version Questionnaire

ተ.ቁ	መጠይቆች	መልስ	መዝለል
**ክፍል አንድ: መለያ**			
101	የሆስፒታል ስም	ሀ. አርባ ምንጭ አጠቃላይ ሆስፒታል<br>ለ. ጨንቻ መጀመሪያ ደረጃ ሆስፒታል<br>ሐ. ሳዉላ አጠቃላይ ሆስፒታል	
102	የሆስፒታል ኮድ	___/___/___	
103	መጠይቁን መሙላት የተጀመረበት ቀን	___/___/___	
104	ወርዳ	________________	
105	ቀበሌ	________________	
106	ሰፍሪ/ጎጥ	________________	
107	ስልክ ቁጥሪ		
108	በአቅራብያ ያለ ጤና ጣብያ		
109	በአቅራብያ ጤና ከላ		
110	የጤና ኤክስተንሰሽ ስምና ስልክ		
111	ሆስፒታል የገቡበት ቀን	____/___/___ዓ.ም	
112	የሆስፒታል ካሪድ ቁጥር	____________	
113	ሆስፒታል እንድመጡ የሪገዉ ማን ነዉ	ሀ. በራሰ ተነሳሽነት<br>ለ. ከለላ ጠና ጣብያ ተልከ	
114	በምን ትራስፖሪት ነዉ የመጣሽዉ	ሀ. በአምቡላስ<br>ለ. በህዝብ ትራስፖሪት<br>ሐ. በግል መከና<br>መ. ለላ, ይጠቀሰ_______	
**ክፍል ሁለት: የነባራዊ ሁኔታ ጥያቀዎች**			
201	የእናት እድመ	________(በሙሉ አመተ)	
202	የጋብቻ ሁኔታ	ሀ .ባለ ትዳሪ<br>ለ. ያላገባች<br>ሐ. ባሏየሞተባት<br>መ. የፈታች<br>ረ. በሲራ ምክንያት የተለያየች	
203	ዘሪ	________________	
204	የእናት የትምህርት ደሪጃ	ሀ. አልተማሪችም<br>ለ. አንደኛ ደሪጃ(1-8)<br>ሐ. ሁለተኛ ደሪጃ(9-12)<br>መ. ኮሌጅና ከዝሃ በላይ	
205	የአባት የትምህርት ደሪጃ	ሀ. አልተማሪም<br>ለ. አንደኛ ደሪጃ (1-8)<br>ሐ. ሁለተኛ ደሪጃ (9-12)<br>መ. ኮሌጅና ከዝያ በላይ	
206	ሀይማኖት	ሀ. አሪቶዶሰ<br>ለ. ካቶልከ	

		ሐ. ፕሮተስታንት<br>መ. ሙስሊም<br>ረ. ባህልዊ	
207	የእናት የስራ ሁኔታ	ሀ. የቤት እመቤት<br>ለ. ነጋደ<br>ሐ. የመንግስት ሰራተኛ<br>መ. የጉልበት ሰራተኛ	
208	የአባት የስራ ሁኔታ	ሀ. አረሶ አደሪ<br>ለ. ነጋደ<br>ሐ. የመንግስት ሰራተኛ<br>መ. ሽማኔ<br>ረ. የጉልበት ሰራተኛ	
209	መነኖሪያ ቦታ	ሀ. ከተማ<br>ለ. ገጠሪ	
210	በአማካይ በወር ምን ያከል ግብ አላቹ?	ሀ. አዉ አለ<br>ለ. የለም	

**ክፍል ሶስት፡ የእረጊዝና እና የወልድ ጊዜ ሁኔታዎችና የምመጡ ችግሮችን በተመለከተ መጠይቅ**			
301	ስንት ጊዜ አረጊዘሻል?	__________(በቁጥር) የመጀመሪያ ከሆነ→	**401**
302	ስንት ጊዜ ወልደሻል?	___________ (በቁጥር)	
303	ሞተዉ የተወለዱ ስንት ናቸዉ?	___________	
304	ዉረጃ አጋጥሞሻል?	ሀ. አዎ<br>ለ. አይ	
305	ያሁኑ አረጊዝናና የቀድሞ በማሃላቸዉ ስንት ወር ልዩነት አለ?	___________ (በወር)	
306	በህይውት ከተወለዱ በሀላ የሞቱ ስንት ናቸዉ?	ሀ. አዎ<br>ለ. አይ	
**ክፍል አራት፡ የእናቶች ሁኔታ ላይ መጠይቅ**			
401	ለዝህ እረጊዝና ቅድመ ወልድ ክትትል አድረገሻል?	ሀ. አዎ<br>ለ. አላደረኩም→	**403**
402	ከላይ ላለዉ ጥያቀ መልሱ አዎ ከሆነ፤ ስንት ጊዜ አድረገሻል?	________________	
403	ድህረ ወለድ ክትትል አለሽ?	ሀ. አዎ<br>ለ. የለኝም	
**ክፍል አምስት፡ በወልድ ጊዜ ሁኔታዎችና የምመጡ ችግሮችን በተመለከተ መጠይቅ**			
501	የወልድሽዉ ቀን?	____/___/_____	
502	በትኛዉ የአላለድ አይነት ነዉ የወለድሽዉ?	ሀ. በትክክለኛዉ የወልድ መንገግ<br>ለ. በስኤስ<br>ሐ. መሳረያ ታጊዘዉ	
503	ደም መፍሰስ አጋጥሞሻል?	ሀ. አዎ<br>ለ. አላጋጠመኝም →	**505**
504	ከላይ ላለዉ ጥያቀ መልሱ አዎ ከሆነ፣ በምን አይነት?	ሀ. ፒላሰንታ ፒረቭያ<br>ለ. ፒላሰንታ አብራሽሽን<br>ሐ. ፒላሰንታ አክራታ/እንክራታ/ፐረክራታ<br>መ. ረትሮ ፒላሰንታል ሄማቶማ<br>ረ. ለላ የመጀመሪያ ትራይምስተረ የደም መፍሰስ	

		ሸ. በወልድ ጊዜ ደም መፍሰስ (ያልተለየ)<br>ቀ. የማፀን መተረተረለላ<br>በ. ከወልድ ቦሃላ ደም መፍሰስ (ያልተለየ)<br>ቸ. ለላ ደም መፍሰስ (ይጠቀስ)______	
505	የሽንት ዉሃሽ የፈሰሰዉ መቸ ነዉ?	ሀ. ምጡ ከመጀመሩ በፍት<br>ለ. ምጥ ላይ እያለዉ	
506	የደም ጊፍትሽ ጨምሮ ያዉቃል?	ሀ. አዎ<br>ለ. አላጋጠመኝም →	508
507	ከላይ ላለዉ ጥያቀ መልሱ አዎ ከሆ፤ የትኛዉ አይነት?	ሀ. ፒረ-እከላምሽያ<br>ለ. እከላምሽያ<br>ሐ. ሄልፒ ስንድረም<br>መ. የቆየ የደም ጊፍት<br>ረ. የእረጊዝና ጊዜ ደም ጊፍት	
508	ደም ማነስ አጋጥሞሻል?	ሀ. አዎ<br>ለ. አላጋጠመኝም →	510
509	ከላይ ላለዉ ጥያቀ መልሱ አዎ ከሆነ፤		
	ሀ. "ህሞግሎብን ለቨል (g/dl)"	_____________	
	ለ. አይታወቅም		
510	"እንፈክሽን" አጋጥሞሻል	ሀ. አዎ<br>ለ. አላጋጠመኝም →	512
511	ከላይ ላለዉ ጥያቀ መልሱ አዎ ከሆነ፤	ሀ. ያልተለየ "እንፈክሽን"<br>ለ. "ፑረፑርያል እንዶመትረያተስ"<br>ሐ. "ፓይሎነፍረያተስ"<br>መ. "ሰፍቲሰምያ"<br>ረ. "ፓረቶናይተስ"<br>ሸ. "ፓርታል ሱጥርሽን"<br>ቀ. ወባ<br>በ. ቅጥኝ<br>ቸ. ለላ "ስሰተምክ እንፈክሽን"	
512	ምጥሽ አስቸግሮሽ ነበረ?	ሀ. አዎ<br>ለ. አይደለም →	514
513	ከላይ ላለዉ ጥያቀ መልሱ አዎ ከሆነ፤	ሀ. "ዩትረያን ፒር ራፒቸር"<br>ለ. "ፕሮሎንግድ ለበር"<br>ሐ. "ፊቶፕልቭክ ድስፒሮፖርሽን"	
514	ለላ "ፓቶጅን"	ሀ. አለ<br>ለ. የለም →	516
515	ከላይ ላለዉ ጥያቀ መልሱ አዎ ከሆነ፤	ሀ. "ቫይረስ"<br>ለ. "እቦልክ ድዝዝ"<br>ሐ. የልብ በሽታ<br>መ. "ስክል ሰል ድዝዝ"<br>ረ. ለላ(___________)	
	**የጨቅላ ህፃናት ሁኔታዎችና እና ችግሮች**		

516	ፐርዘንተሽን	ሀ. “ሰፋልክ”<br>ለ. “ብረች”<br>ሐ. “ትራስቨረስ/ፈስ/ብሮዉ<br>መ. ለላ(____________)	
	የጨቅላ ህፃኑ ፆታ?	ሀ. ወንድ<br>ለ. ሴት	
	“በረዝ ትሮዉማ”	ሀ. አለ<br>ለ. የለም	
	ከላይ ላለዉ ጥያቀ መልሱ አዎ ከሆነ፤	___________________	
	ጨቅላ ህፃኑ ለላ ህከምና ቦታ ተልኮ ነበር?	ሀ. አለ<br>ለ. የለም	
	የጨቅላ ህፃን ጽኑ ህሙማን ግብቶ ነበር	ሀ. አዎ<br>ለ. አይደለም	

እሄ ቅፅ የምሞላዉ ጨቅላ ህፃኒ ከሞተ ብቻ ነዉ፡፡ ለዝህ ቅፅ መልስ ልመልስ የምችለዉ ጤና ባለሞያ ወይም ከሞተዉ ጨቅላ ህፃን ጋረ ዝምድና ያለዉ ሰዉ ብሆን ይመረጣል፡፡

**ክፍል ስባት፡-ቅፅ ሁለት (ጨቅላ ህፃኑ ከሞተ ብቻ ይሞላ)**		
**601. ህጻኑ ሞትዋል?**	*ሀ. አዎ*<br>*ለ. አልሞተም* →	**አቁም**

የስምምነት ቅፅ

ጤና ይስጥልኝ! ስሜ____እኔ የምሰራዉ አረባ ምንጭ ዩኒቨረስት ነዉ፡፡ እኛ የምንሰበስብዉ መረጃ ህይዎታቸዉ ያለፈዉ ጨቅላ ህፃናት ህይዎታቸዉ እንድያፍ ያረጉት ምክንያቶችን በተመለከተ ነዉ፡፡ ስለዝህ በመሳተፎ በጣም እናመሰግናለን፡፡ እንኛ የምንጠይቀዉ ጨቅላ ህፃናት እንድሞቱ ላረጉ ሁኔታዎችን በተመለከተ ነዉ፡፡ ከረሶ የምሰበሰበዉ የትኛዉም አይነት መረጃ በምስጥረ ይጠበቃል፡፡ እረሰዉም ሆነ የሞተዉ ጨቅላ ህፃን በተመለከተ የተሰበሰበዉ መረጃ በትኛዉም አይነት ምክንያት ከመረጃ ሰብሳብዎች ዉጭ አይሰጥም፡፡

በዝህ ጥናት መሳተፍ በፍላጎት ስሆን ለአንድ አንድ ጥያቀዉችም ሆነ ለሁሉም ጥያቀዎች አለመመለስ ሙሉ መፍት አለዎት፡፡ መጠይቁን በሙሉ በትኛዉም ሰዓት ማቆምም ሆነ አለመመለስ ይቻላል፤ ማቆሞ ምንም ነገረ ልያስከትሎ አይችልም፡፡ ነገረ ግን የረሶ መሳተፍ ለዝህ ጥናት መሳካት ትልቅ ምና መኖሩና በጤና ተቋማት የምሰጡ አግልግሎቶችን ለማስተካከል ጥሩ ግባዓት መሆኑን በአፅኖት እንገልፃለን፡፡

በዝህ ሰዓት ስለዝህ መጠይቅ ጥቅምና የተካተቱ ነግሮችን መጠየቅ ይፈልጋሉ?

አሁን መጠይቁ መጀመረ እችላለዉ?

የመረጃ ሰባሳብዉ ፊረማ፡____________________________ ቀን፡__________________

ተጠያቅዉ ከተስማማ **ይቀጥሉ** ካልተሰማማ **ያቁሙ**

602	በዝህ ሰዓት እናቷ/ቱ በህይዎት አለች?	*ሀ. አዎ*<br>*ለ. የለችም*	
603	መላሹ ጤና ባለሞያ ነዎ?	*ሀ. አዎ* →<br>*ለ. አይደለም*	**605**
604	ከላይ ላለዉ ጥያቀ መልሱ አይደልም ከሆኔ ከሞተዉ ጨቅላ ህፃን ጋራ ምን አይነት ግንኙነት ነዉ ያለዉ?	ሀ. እናት<br>ለ. አባት<br>ሐ. የወንድ አያት<br>መ. የሴት አያት<br>ረ. አጎት<br>ሸ. አክስት<br>ቀ. ለላ (ይጠቀስ) ____________	
605	ጨቅላ ህፃኑ የት ነዉ የሞተዎ?	ሀ. ሆስፒታል<br>ለ. ጤና ጣብያ<br>ሐ. ጤና ኬላ<br>መ. ወደ ጤና ተቋም እየሄደች<br>ረ. ቤት<br>ሸ. ላለ(ይጠቀስ)__________	
606	ህይዎቱ ያለፈዉ ጤና ተቋም ከሆኔ የጤና ተቋሙ ስሙና አድራሻዉ ይፃፍ?	የተቋሙ ስምና አድራሻ_______<br>የገባበት ቀን__________	

<u>በማብራራት የምገለፅ የታረክ ጥያቀ</u>

607. የጨቅላ ህፃኑ የህመም ሁኔታዉና ለሞት ያበቃዉ ነገረ ማባራራት ይችላሉ?

ለጠያቅዉ ትዛዝ፡ መላሹ የጨቅላ ህፃኑ የህመም ሁኔታዉና ለሞት ያበቃዉ ነገረ በራሱ ቃል እንድያብራራና ተጨማረ ሃሳባ እድያዎራ ከመጠየቅ በስተቀረ ማዎጣጣት እንደማይቻል ማዎቅ አለበት፡፡

____

____

____

____

______________________________<br>______________________________<br>______________________________<br>______________________________<br>______________________________<br>608. ጊዜ ከወሰዱ ቦሃላ መላሹ ስያብራረ ከሰጠዉ ዉስጥ ካለ ከምከተሉት ያክብቡ፡፡ ለቀሩት ጥያቀዉች የምከተለዉን እንደ መረህ ይጠቀሙ፡፡<br>1. ተቅማጥ 2. ሳል 3. ትኩሳት 4. ሽፍታ 5. ጉዳት 6. ኤራስን መሳት 7. ፊት 8. የማጅራት ግትረ 9. ተታነስ 10. ኩፍኝ 11. ካሸረኮረ 12. ማረስመስ 13. የመተነፍስ ችግረ 14. ቶሎ ቶሎ መተንፈስ 15. የተወሳሰበ ወለድ 16. ማልፈረመሽን 17. በጣም ትንሽ ህፃን 18. በጣም ቀጭን ህፃን 19. ጊዜዉ ሳይደረስ የተወለደ 20.ንሞንያ 21. ወባ 22. ጃወንድስ 23.ለላ (ይጠቀስ) ____________			
609	ህፃኑ ከሞሞቱ በፊት ምን ያክል ጊዜ ታሚል?	_______(በቀን)	
610	ህፃኑ ስታመም ከቤት ዉጭ ታከመዉ(ተንከባከበዉ) ያዉቃል?	ሀ. አዎ<br>ለ. አያዉቅም →	**612**
611	ከላይ ላለዉ ጥያቀ መልሱ አዎ ከሆኔ፣ የት ነዉ የተንከባከበዉ? (ከአንድ በላይ መልስ ይቻላል፤ የምከተሉት መሆናቸዉን ያወጣጡ)<br>1. የመንግስት ሆስፒታል<br>2. የመንግስት ጤና ጣብያ<br>3. የመንግስት ጤና ኬላ	ሀ. የመንግስት ሆስፒታል<br>ለ. የመንግስት ጤና ጣብያ<br>ሐ. የመንግስት ጤና ኬላመ.<br>መ. የግል ክልንክ<br>ረ. የግል ፓረማስ፣ የመህዳንት መስጫ<br>ሸ. ለላ(ይጠቀስ)________	
612	የሞተዉ ህፃን ስወለድ የአካል ችግረ አለበት?	ሀ. አዎ<br>ለ. የለበትም →	**614**
613	ከላይ ላለዉ ጥያቀ መልሱ አዎ ከሆኔ፣ የምን አይነት ችግረ?<br><br>(ከአንድ በላይ መልስ ይቻላል)	ሀ. የጭንቅላት<br>ለ. የሰዉነት<br>ሐ. የእጅ/የእጅ የመዳፍ<br>መ. የእግረ/የእግረ መዳፍ<br>ረ. ለላ (ይጠቀስ)_________________	
614	ህፃኑ ከተወለድ ቦሃላ ስተነፍስ ነበረ?	ሀ. አዎ<br>ለ. አይተነፍስም	
615	ህፃኑ ከተወለድ ቦሃላ ጡት ወይም ጡጦ ስስብ ነበረ?	ሀ. አዎ<br>ለ. አይስብም/አይጠባም	
616	ህፃኑ ጡት መሳብ ያቆመዉ በትክክል ከጀመረ ቦሃላ ነዉ?	ሀ. አዎ<br>ለ. አይደለም →	**618**
617	ከላይ ላለዉ ጥያቀ መልሱ አዎ ከሆኔ፣ ከሞሞቱ በፍት ምን ያክል ጊዜ አቁሞ ነዉ?	ሀ. ከ1 ቀን በታች<br>ለ. ከ1-2 ቀን<br>ሐ. ለ2 ቀን በላይ	
618	ከላይ በተ.ቁ 716 ላለዉ ጥያቀ መልሱ አዎ ከሆኔ፣ ከተወለደ በስንተኛ ቀኑ ነዉ ጡት መሳብ ያቆመዉ?	ሀ. ከ1 ቀን በታች<br>ለ. ከ1-2 ቀን<br>ሐ. ከ3-7 ቀን<br>መ. ከ8-14 ቀን<br>ረ. ከ15-28 ቀን	

619	ህፃኑ ስወለድ ጮሆ ነበረ?	ሀ. አዎ<br>ለ. አልጮሄም	
620	ህፃኑ ከተወለድ ቦሃላ መጮሁን አቁሞ ነበረ?	ሀ. አዎ<br>ለ. አላቆመም → 622	
621	ከላይ ላለዉ ጥያቀ መልሱ አዎ ከሆኔ፣ ከሞሞቱ በፍት ጡት መሳብ ምን ያክል ጊዜ አቁሞ ነዉ?	ሀ. ከ1 ያነሰ<br>ለ. አንድ ቀንና ከዝሃ በላይ	
622	ህፃኑ ህይወቱ እንድያፍ ያረገዉን ህመም ስታመም እስፓዝም/ኮንቨልዥ አለበት?	ሀ. አዎ<br>ለ. የለበትም	
623	ህፃኑ ህይወቱ እንድያፍ ያረገዉን ህመም ስታመም እራሱን ስቶ ነበረ?	አ. አዎ<br>ለ. አልሳታም	
624	ህፃኑ ህይወቱ እንድያፍ ያረገዉን ህመም ስታመም በልጂድ ፎንታለን አለበት?	ሀ. አዎ<br>ለ. የለበትም	
625	ህፃኑ ህይወቱ እንድያፍ ያረገዉን ህመም ስታመም ቴታነስ አለበት?	ሀ. አዎ<br>ለ. የለበትም	
626	ህፃኑ ህይወቱ እንድያፍ ያረገዉን ህመም ስታመም አይኑ ወደ ብጫ ተቀይሮ ነበረ?	ሀ. አዎ<br>ለ. አልተቀየረም	
627	ህፃኑ ህይወቱ እንድያፍ ያረገዉን ህመም ስታመም የብረት ጫፍ ላይ መቅላት ወይም ፈሳሽ ነበረበት?	ሀ. አዎ<br>ለ. የለበትም	
628	ህፃኑ ህይወቱ እንድያፍ ያረገዉን ህመም ስታመም የሰዉነት መሞቅ ወይም መቅላት አለበት?	ሀ. አዎ<br>ለ. የለበትም	
629	ህፃኑ ህይወቱ እንድያፍ ያረገዉን ህመም ስታመም በቆዳ ላይ ሽፍታና መግል የቋጠረ ነገረ ነበረ?	ሀ. አዎ<br>ለ. የለዉም	
630	ህፃኑ ህይወቱ እንድያፍ ያረገዉን ህመም ስታመም ትኩሳት ነበረበት?	ሀ. አዎ፣ ለ____ቀን የቆየ<br>ለ. የለዉም	
631	ህፃኑ ህይወቱ እንድያፍ ያረገዉን ህመም ስታመም ተቅማጥ ነበረበት?	ሀ. አዎ፣ ለ____ቀን የቆየ<br>ለ. የለዉም	
632	ህፃኑ ህይወቱ እንድያፍ ያረገዉን ህመም ስታመም ሳል አለዉ?	ሀ. አዎ፣ ለ____ቀን የቆየ<br>ለ. የለዉም	
633	ህፃኑ ህይወቱ እንድያፍ ያረገዉን ህመም ስታመም የመተንፈስ ችግረ ነበረዉ?	ሀ. አዎ፣ ለ____ቀን የቆየ<br>ለ. የለዉም	
634	ህፃኑ ህይወቱ እንድያፍ ያረገዉን ህመም ስታመም ቶሎቶሎ ስተንፍስ ነበረ?	ሀ. አዎ፣ ለ____ቀን የቆየ<br>ለ. አይደለም	
635	ህፃኑ ህይወቱ እንድያፍ ያረገዉን ህመም ስታመም ለብዙ ሰዓት	ሀ. አዎ<br>ለ. የለዉም	

	መተንፍስ አቁመዉ እህፃኑ ንደግና የጀመረበት ሁኔታ ነበረዉ?		
636	ህፃኑ ህይወቱ እንድያፍ ያረገዉን ህመም ስታመም ቾስት እንድረወግ ነበረዉ?	ሀ. አዎ<br>ለ. የለዉም	
637	ህፃኑ ህይወቱ እንድያፍ ያረገዉን ህመም ስታመም የአፍንጫ ጫፍ መንቀሳቀስ ነበረዉ?	ሀ. አዎ<br>ለ. የለዉም	
638	ህፃኑ ህይወቱ እንድያፍ ያረገዉን ህመም ስታመም ንሞንያ(የብረድ በሽታ) ነበረዉ?	ሀ. አዎ<br>ለ. የለዉም	
639	የህፃኑ እናት ኤችአይቭ ተመረምራለች?	ሀ. አዎ<br>ለ. አለተመረመረችም →	**አቁም**
640	ከላይ ላለዉ ጥያቀ መልሱ አዎ ከሆኔ፣ ዉጠቱ ፖዘትቭ ነበረ?	ሀ. አዎ<br>ለ. አይደለም	
641	የህፃኑ እናት በጤና ባለሞያ ኤድስ አለብሽ ተብላ ታዉቃለች?	ሀ. አዎ<br>ለ. አታቅም	

አመሰግናለሁ !!!
