# Supplementary material for: Neonatal mortality and its determinates in public hospitals of Gamo and Gofa zones, southern Ethiopia: prospective follow up study
Source: BMC Pediatr. 2019 Dec 16;19:499. doi: 10.1186/s12887-019-1881-0 (PMC6912940; doi:10.1186/s12887-019-1881-0)
Supplement: Supplementary file 2 — Additional file 2. STROBE checklist [file 12887_2019_1881_MOESM2_ESM.pdf]

STROBE Statement—checklist of items that should be included in reports of observational studies

	Item No.	Recommendation	Page No.	Relevant text from manuscript
**Title and abstract**	1	(*a*) Indicate the study's design with a commonly used term in the title or the abstract	-	**Neonatal Mortality and Its Determinates in Public Hospitals of Gamo and Gofa Zones, Southern Ethiopia: Prospective Follow up Study**
		(*b*) Provide in the abstract an informative and balanced summary of what was done and what was found	-	**Background:** The neonatal period is the most vulnerable time for child survival. The declines in the neonatal mortality rate have been slower than the post-neonatal under-five mortality rate in the majority of countries. This trend is also similar in Ethiopia, that neonatal mortality was high as compared to the post-neonatal mortality rate. A large proportion of neonatal deaths occur during the 48 hours after delivery. Different studies were conducted in assessing determinates for neonatal mortality but there is a need to assess the immediate postnatal (within two days following delivery) cause of neonatal mortality that the majority of deaths occurred at that time. So, this study is to fill those gaps of the aforementioned studies, in assessing the determinate factors affecting neonatal mortality in public hospitals of Gamo and Gofa Zones, Southern Ethiopia.<br><br>**Methods:** A prospective follow up study was conducted among 6,986 study participants from April 5, 2018, to March 5, 2019. All live births at the hospitals during the study period were included in this study. Data on causes of neonatal death were collected by using a structured verbal autopsy questionnaire. Data were entered into Epi data version 3.1 and exported to Stata version 15 for analysis. Crude and adjusted estimate β with 95%CI was calculated in the binary logistic regression model. The goodness of fit was tested by a log-likelihood ratio (LR). In this study P-value < 0.05 was considered to declare a result as a statistically significant association.<br><br>**Results:** In this study, neonatal mortality incidence ratio was 9.6 (95%CI: 7.5, 12.2) per 1000 live births. Age of the mother, number of antenatal care, hemorrhage, sex of the neonate, presentation, gestational age and birth weight were identified as the significant determinates for neonatal mortality cases. Prematurity, infection, and birth asphyxia were the most common causes of neonatal mortality cases.<br><br>**Conclusions:** This study indicated that a significant number of neonates died during the neonatal period. Both maternal and neonatal factors were identified. Therefore, early identification of obstetric complications and immediate

				interventions, strengthening the provision of quality antenatal and postnatal care services are recommended.<br><br>***Keywords:*** *Neonatal Mortality; Neonatal Deaths; Gamo and Gofa Zones*
**Introduction**				
Background/rationale	2	Explain the scientific background and rationale for the investigation being reported	1	**Background**<br>Globally, there is impressive advancements have been made on many health fronts from 2000 to 2017. However, to meet the Sustainable Development Goals' health targets by 2030, progress must be accelerated, in particular regions with the highest burden of disease [1]. The third Sustainable Development Goals (SDG3) aimed to end preventable deaths of newborns and reduce neonatal mortality to at least as low as 12 per 1000 live births in all countries [2].<br>Despite all efforts to decrease neonatal mortality, recent data show that neonatal mortality has declined at a slower rate than overall childhood mortality, which has resulted in neonatal mortality now accounting for 46% of overall under-five childhood deaths [3]. The neonatal mortality is 18 globally, and 26.7 in Africa in 2017, and 30 in Ethiopia per 1000 live births in 2019[4, 5]. Ethiopia Mini-Demographic Health Survey, 2019 indicated that there is a slight increase in neonatal mortality, and it was high as compare to the post-neonatal mortality rate. A large proportion of neonatal deaths occur during the 48 hours after delivery, and these first two days following delivery are critical for monitoring complications arising from the delivery [4, 6].<br>The period around birth constitutes a critical window of opportunity for the prevention and management of maternal and newborn complications, which can otherwise prove fatal. A large "proportion of" newborn illnesses and deaths can also be prevented using simple, low-cost interventions during delivery and the week following partum[7]. Reducing neonatal mortality is increasingly important not only because deaths that occur during the neonatal period is increasing as under-five mortality declines but also health interventions needed to address the major causes of neonatal deaths generally differ from those needed to address other under-five deaths [8]. A significant proportion of these neonatal deaths could be prevented by the appropriate management of the neonate presenting complications, such as very low birth weight, < 30 gestational weeks at birth or an Apgar score at the 5th minute of life <7[9].<br>Analysis of different studies in Ethiopia showed that the incidence of the neonatal mortality rate was ranged from 17.2 to 35.5 per 1000 live births [10-15]. The most determinate factors which were identified by previous studies were birth order, frequency of antenatal care, delivery place, twin delivery and size of neonate [10, 16, 17]. Birth asphyxia, neonatal infections, and prematurity were the three leading causes of neonatal mortality [10, 11].

				Newborns in Ethiopia gaining attention through the Global Maternal Child Survival Program: Contributes to reductions of neonatal morbidity and mortality through capacity-building in high-impact services both at the community and the primary health care unit levels. The activity supports the government of Ethiopia to improve community maternal and newborn health practices and care-seeking behaviours; increases the provision of quality community-based newborn care services including management of newborn sepsis; and strengthens the supportive systems with a focus of district capacity building [18]. This program is underway, but to scale up a comprehensive way of implementation identifying determinate factors intensively is very important to reduce neonatal mortality further. Different studies were conducted in assessing determinates for neonatal mortality but there is a need to assess the immediate postnatal (within two days following delivery) cause of neonatal mortality that the majority of deaths occurred at that time. Therefore, there is a need for research in public hospitals of Gamo and Gofa Zones to assess the incidence, underlying causes and determinate factors for neonatal mortality.
Objectives	3	State specific objectives, including any pre-specified hypotheses	-	To estimate the incidence of neonatal mortality in public hospitals of Gamo and Gofa Zones, Southern Ethiopia<br>To identify the determinates for neonatal mortality in public Hospitals of Gamo and Gofa Zones, Southern Ethiopia
**Methods**				
Study design	4	Present key elements of study design early in the paper	2	A prospective follow up study design was employed.
Setting	5	Describe the setting, locations, and relevant dates, including periods of recruitment, exposure, follow-up, and data collection	2	This prospective follow up study was conducted in public hospitals of Gamo and Gofa Zones from April 5, 2018, to March 5, 2019. There are six hospitals in Gamo and Gofa Zones but this study was done in selected three public hospitals (Arba Minch General Hospital (AMGH), Sawla General Hospital (SGH) and Chencha Primary Hospital (CPH)). The total population of the study area is 2,019,687. The estimated number of women of reproductive age (15-49) is 470,587 from this, the estimated number of delivery is 69,881 and the estimated number of live birth is 69,881. In Gamo and Gofa Zone, the institutional skilled delivery rate is 51.2% [19, 20].<br>A structured interviewer-administered pre-tested questionnaire and standard abstraction checklist to review data from medical records were used to collect the data. The tools were developed adapted by reviewing different works of literature. Data on causes of neonatal death were collected by using a structured verbal autopsy (VA) questionnaire adapted from the standard VA questionnaire developed and validated by WHO, Johns Hopkins University (JHU) and London School of Hygiene and Tropical Medicine [21].<br>Neonates who experienced mortality cases during the follow up period were identified prospectively by trained six BSc holder midwives and supervised by two MSc holder nurses. As this was a prospective follow-up study; data were collected

				in different phases: In the first phase: all the baseline information in the hospital was collected either by interviewing or by abstracted from medical records. The data were collected from the delivery ward, postnatal ward and neonatal intensive care unit (NICU) of each hospital. For the neonates that died in the hospital stay, VA was conducted at a point in time. But, for those neonates who survived in the hospital stay the second phase proceeded at the end of the neonatal period. So, newborns were assessed for mortality cases whether they died within 28 days of life or survived and for those who don't survive VA was conducted.
Participants	6	(*a*) *Cohort study*—Give the eligibility criteria, and the sources and methods of selection of participants. Describe methods of follow-up<br>*Case-control study*—Give the eligibility criteria, and the sources and methods of case ascertainment and control selection. Give the rationale for the choice of cases and controls<br>*Cross-sectional study*—Give the eligibility criteria, and the sources and methods of selection of participants	2	All live births in three selected hospitals of Gamo and Gofa Zones
		(*b*) *Cohort study*—For matched studies, give matching criteria and number of exposed and unexposed<br>*Case-control study*—For matched studies, give matching criteria and the number of controls per case		Not applicable
Variables	7	Clearly define all outcomes, exposures, predictors, potential confounders, and effect modifiers. Give diagnostic criteria, if applicable	3	Neonatal mortality case was the dependent variable and socio-demographic and economic characteristics, maternal factors, maternal and child health services and obstetric factors were independent variables for this study.
Data sources/ measurement	8*	For each variable of interest, give sources of data and details of methods of assessment (measurement). Describe comparability of assessment methods if there is more than one group	3	Death of neonates before 28 days of neonatal life after developed life-threatening condition [21].<br>A neonate who fulfilled standard definition and appropriate criteria and died was categorized as mortality cases and coded as "1" and who survived was coded as "0".
Bias	9	Describe any efforts to address potential sources of bias		Maximum efforts was done to decrease biases (selection bias, information bias, even recall bias for verbal autopsy). All the live births were included, standard and validated tools were used. Experienced data collectors, and supervisors were used to collect the data, cross-checking of different sources was used to confirm the validity of the data to be collected. Proper coding, and categorization of data were maintained.
Study size	10	Explain how the study size was arrived at<br><br>Continued on next page	2	Sample size for this study was estimated by using Epi info7 software Stat Cal. The sample size was calculated by taking the most determinate factors for neonatal mortality; which was gestational age at birth from the study pervious conducted in Southwest Ethiopia [10]. Based on this, the prevalence of neonatal mortality among unexposed group (gestational age greater than 37 weeks) was 2.9% (p1=0.029) and the prevalence of neonatal mortality among exposed group (gestational age less

				than 37 weeks) was 5.8 (p2=0.058) and by considering 95% level of confidence, power of 90 and ratio of 1:1. So, the calculated sample for this study was 2433 after adding a non-response rate of 10%. But, the sample size used for this study was 6,986 based on the number of live births in the respective hospitals in one year period.
Quantitative variables	11	Explain how quantitative variables were handled in the analyses. If applicable, describe which groupings were chosen and why	3	Frequency, percentage, summery statistics (mean and SD), and recoding was done
Statistical methods	12	(*a*) Describe all statistical methods, including those used to control for confounding	3	Data were coded, cleaned, edited and entered into Epi data version 3.1 and then exported to Stata version 15 for analysis. Binary logistic regression was done to see the association between each independent variable and outcome variable. The goodness of fit was tested by a log-likelihood ratio (LR). All variables with P<0.25 in the bivariate analysis were included in the final model of multivariable analysis in order to control all possible confounders. Variance inflation factor (VIF) >10 and Tolerance (T) <0.1 were considered as suggestive of the existence of multi co-linearity. A crude and adjusted Beta (β) coefficient with 95%CI was estimated in order to identify determinates for the neonatal mortality cases. In this study P-value < 0.05 was considered to declare a result as a statistically significant association.
		(*b*) Describe any methods used to examine subgroups and interactions	3	Not applicable
		(*c*) Explain how missing data were addressed	3	Data were checked for completeness, accuracy, clarity, and consistency before data entry into the software. After entering, multiple imputation technique was used to iterate some missed data.
		(*d*) *Cohort study*—If applicable, explain how loss to follow-up was addressed<br>*Case-control study*—If applicable, explain how matching of cases and controls was addressed<br>*Cross-sectional study*—If applicable, describe analytical methods taking account of sampling strategy	3	For all study participants: address of them, address of the surrounding health facilities, address of health extension workers were registered before discharge from the health care institution, and maximum effort was invested to trace loss to follow ups.
		(*e*) Describe any sensitivity analyses	-	Not applicable
**Results**				
Participants	13*	(a) Report numbers of individuals at each stage of study—eg numbers potentially eligible, examined for eligibility,	3	In this study, 6986 study participants were interviewed in the baseline after excluding 131 twin deliveries from total live births in three selected public hospitals from two zones of Southern Ethiopia. During follow up for 28 days 153

		confirmed eligible, included in the study, completing follow-up, and analysed		study participants became lost to follow up and 64 were excluded from the study because of inconsistent and incomplete information. At the end of follow up, 6769 study participants stayed in the cohort and interviewed the end line which gave a response rate of 96.9%. During follow up 6704 neonates were survived and 65 died. A verbal autopsy had conducted among 52 died neonates and the rest were refused.
		(b) Give reasons for non-participation at each stage		The mean reason for non-participation (loss to follow up) were changing study area, and unwillingness of some study participants.
		(c) Consider use of a flow diagram		For the diagram; (See figure 1 from main manuscript)
Descriptive data	14*	(a) Give characteristics of study participants (eg demographic, clinical, social) and information on exposures and potential confounders	4	**Socio-demographic and economic characteristics of the respondents**<br>Of the neonate's mother, 3384 (50.0%) were age ranged 25-34 years old and with mean and standard deviation (SD) of 25.3±5.02. The majority (95.0%) were married and 3727 (55.0%) had from Gamo ethnicity group. Regarding the educational status of neonates mother, 1439 (21.3) had no formal education and 2069 (30.6%), 1822 (26.9%) and 1439 (21.3%) had primary (grade 1-8), secondary (grade 9-12) and college and above respectively. Two thousand eighty (30.7%) of the neonate's father had the educational status of college and above and 2497 (36.9%) had merchant. Out of neonates mother 2885 (42.6%) had Orthodox religion follower and 420 (6.2%), 2966 (43.8%), 451 (6.7%) and 47 (0.7%) were Catholic, Protestant, Muslim and traditional respectively. More than half (57.7%) of the neonates mother was housewife and 1057 (15.6%), 1403 (20.7%), 123 (1.8%) and 283 (4.2) were merchant, government employer, daily labor and student respectively and 4067 (60.1%) had urban residents (Table 1).

Table 1: Socio-demographic and economic characteristics of study participants in public hospitals of Gamo and Gofa Zones, Southern Ethiopia, 2018/9 (*n=6769*)

Characteristics	Frequency	Percentage
**Age**		
15-24	3002	44.3
25-34	3384	50.0
≥35	383	5.7
**Marital status**		
Married	6430	95.0
Other*	339	5.0
**Ethnicity**		
Gamo	3725	55.0
Gofa	1519	22.4
Other†	1525	22.6
**Educational status of the father**		
No formal education	1026	15.2

Primary (1-8)	1636	24.2
Secondary (9-12)	2027	29.9
College and above	2080	30.7
**Occupation of the father**		
Farmer	1773	26.2
Merchant	2497	36.9
Government employer	1974	29.2
Wavier	275	4.1
Daily laborer	250	3.7
**The average income per month**		
<70.8USD	1775	26.2
70.8-177USD	3195	47.2
>177USD	1799	26.6

single, divorced and separated due to work

†Zayise, Amhara, Oromo, Gurage, Woliata, Konso, Derashe, Oyida, and Gidicho

Maternal and child health, and obstetric factors

Out of the neonate's mother 3900 (57.6%) had multipara (birth order ≥2), only 350 (9.0%) had a history of the stillbirth and 434 (11.1%) encountered loss of conceptus. Two thousand eight hundred (71.8%) of the mothers of the neonates were birth inter of 24-48 month and 329 (8.4%) had a history of neonatal death. Of the neonate's mothers, 6004 (88.7%) had antenatal care (ANC) and 6674 (98.6%) had immediate postnatal care. Regarding mode of delivery 4943 (73.0%) gave birth by spontaneous vaginal delivery, 243 (3.6%) were instrumental and 1583 (23.4) were by caesarean section. One thousand two hundred sixty-two (18.6%) encountered premature rupture of membrane and 524 (7.7%) developed hypertension (HTN) during pregnancy. Out of the neonate's mothers, 193 (2.9%) had anemic and 682 (10.1%) faced dystocia. From those who faced labor dystocia, 24 (3.5%) had due to uterine pre-rupture, 465 (68.2%) had due to prolonged labor and 193 (28.3%) had due to feto-pelvic disproportion. Two hundred thirty (3.4%) of the neonate's mothers encountered infection and 130(1.9%) had developed other pathologies. Of the mothers who developed infection 33 (14.4%) had an unspecified infection and 50 (21.7%), 100 (43.5%) and 47 (20.4%) had puerperal endometritis, pyelonephritis and others (syphilis and malaria) respectively. From the mothers who developed other pathologies 33 (25.4%) had HIV/AIDS, 58 (44.6%) had heart diseases and 39 (0.3%) had others (DM, thyroid disorder, embolism, and DIC). Regarding the presentation of neonates, 5818 (86.0%) delivered with vertex and 3606 (53.3%) were male neonates. Of the neonates, 65 (1.0%) encountered birth trauma during delivery. From those 24 (36.9%) of the

neonates had cephalhematoma, 9 (13.8%) developed caput succedaneum and 32 (49.3%) had others (fracture, bruising and subgleal hemorrhage) (Table 2).

Table 2: Maternal and child health and obstetric factors of study participants in public hospitals of Gamo and Gofa Zones, Southern Ethiopia, 2018/9 (*n=6769*)

Variables	Frequency	Percentage
**Number of ANC visit**		
No visit	765	11.3
1-3	1820	26.9
≥ 4	4184	61.8
**Hemorrhage**		
Yes	315	4.7
No	6454	95.3
**Cause of hemorrhage**		
Placenta praevia	108	34.3
PPH	153	48.6
Other®	54	17.1
**Premature rupture of membrane**		
Yes	1262	18.6
No	5507	81.4
**Hypertension during pregnancy**		
Yes	524	7.7
No	6245	92.3
**Classification of HTN**		
Pre-eclampsia	297	56.7
Eclampsia	74	14.1
Chronic hypertension	77	14.7
Gestational hypertension	76	14.5
**Presentation**		
Vertex	5818	86.0
Non-vertex©	951	14.0
**Sex of the neonates**		
Male	3606	53.3
Female	3163	46.7
**Gestational age**		
<37 week	808	11.9
≥37 week	5961	88.1
**Birth weight**		
<2500g	600	8.9
≥2500g	6169	91.1
**Baby referred to other health facilities**		

<table>
<tr><td></td><td></td><td></td><td></td><td>
<table>
<tr><td>Yes</td><td>77</td><td>1.1</td></tr>
<tr><td>No</td><td>6692</td><td>98.9</td></tr>
</table>
®accreta/increta/percreta, hemorrhage during delivery, uterine rupture, and other obstetric hemorrhages, and ©breech, transverse, face, and brow
Causes of neonatal mortality
In this study, 65 neonatal deaths occurred during the follow-up period in selected three public hospitals of Gamo and Gofa Zones, Southern Ethiopia. Of the neonatal deaths, only 52 respondents were agreed and interviewed for verbal autopsies but rest were refused for verbal autopsy. Almost half (51.9%) of the neonatal deaths were happened due to prematurity or gestational age less than 37 week, 13 (25%) due to neonatal infection, 7 (13.5%) were by birth asphyxia, 3(5.8%) congenital malformation due congenital malformation and the rest were with unspecified cause.
</td></tr>
<tr><td></td><td></td><td>(b) Indicate number of participants with missing data for each variable of interest</td><td></td><td>The number of missing data were 25</td></tr>
<tr><td></td><td></td><td>(c) Cohort study—Summarise follow-up time (eg, average and total amount)</td><td>4</td><td>The follow-up period time was neonatal period (28 days)</td></tr>
<tr><td>Outcome data</td><td>15*</td><td>Cohort study—Report numbers of outcome events or summary measures over time</td><td></td><td>
Incidence of neonatal mortality
In this study inter and intra-hospital neonatal mortality incidence ratio was estimated with a 95% level of confidence per 1000 live births. The highest proportion of neonatal mortality was reported from Chencha Primary Hospital that 1.0% (95%CI: 0.5, 2.20%) Overall, neonatal mortality incidence ratio in selected three public hospitals was 0.96% (95%CI: 0.75, 1.22%) (Table 3).
Table 3: Incidence of neonatal mortality among study participants in selected hospitals of Gamo and Gofa Zones, Southern Ethiopia, 2018/9 (n=6769)
<table>
<tr><th>Name Hospital</th><th>n(%) of NM</th><th>Total number of live births</th><th>NMIR® with 95%CI per 1000 live births</th></tr>
<tr><td>AMGH</td><td>42(64.6)</td><td>4455(65.8)</td><td>9.4(6.9,12.7)</td></tr>
<tr><td>CPH</td><td>8(12.3)</td><td>794(11.7)</td><td>10.1(5.0,20.0)</td></tr>
<tr><td>SGH</td><td>15(23.1)</td><td>1520(22.5)</td><td>9.9(5.9,16.3)</td></tr>
<tr><td>Overall</td><td>65(100)</td><td>6769(100)</td><td>9.6(7.5,12.2)</td></tr>
</table>
®Neonatal mortality incidence ratio
</td></tr>
<tr><td></td><td></td><td>Case-control study—Report numbers in each exposure category, or summary measures of exposure</td><td></td><td></td></tr>
</table>

		Cross-sectional study—Report numbers of outcome events or summary measures		
Main results	16	(*a*) Give unadjusted estimates and, if applicable, confounder-adjusted estimates and their precision (eg, 95% confidence interval). Make clear which confounders were adjusted for and why they were included	7	**Determinates of neonatal mortality** After adjusting in the multivariable model age of the mother, the number of ANC visits, hemorrhage, and presentation, gestational age at birth, birth weight and sex of the neonate were significantly associated with neonatal mortality. Advanced maternal age above 35 years old increased neonatal mortality significantly as compared to the age group 15 to 24 years old (β =1.34; 95% CI:0.55,2.14). The number of the antenatal visit from 1 to 3 significantly reduced neonatal mortality as compared to four or more visits (β =-0.80; 95% CI:-1.47,-0.13) and hemorrhage increased neonatal mortality significantly (β =0.95; 95% CI: 0.19, 1.71). Non-vertex presentation (β =1.19; 95% CI: 0.64, 1.74), gestational age of less than 37 week (β =1.17; 95% CI: 0.47, 1.88), birth weight of less than 2500g (β =0.73; 95% CI: 0.01, 1.45) and being male neonate (β =0.90; 95% CI: 0.19, 1.60) had significantly increased neonatal mortality (Table 4).

Table 4: Bivariate and multivariable analysis of determinates for neonatal mortality among study participants in selected hospitals of Gamo and Gofa Zones, Southern Ethiopia, 2018/9 (*n=6769*)

**Variables**	**Crude estimate β 95%CI**	**Adjusted estimate β**
**Place of residence**		
Urban	-0.69(-1.19,-0.20)	-0.35(-0.93,0.23)
**Age of the mother**		
25-34	-0.26(-0.86,0.34)	-0.65(-1.41,0.11)
≥35	2.07(1.47,2.66)	1.34(0.55,2.14)*
**Birth interval**		
Not applicable (primi)	NA	NA
<24 month	-1.34(-2.74,0.06)	-0.89(-2.54,0.74)
24-48 month	-0.66(-1.86,0.53)	-0.43(-1.80,0.95)
**Number of ANC visits**		
No visit	2.12(1.57,2.68)	-0.69(-1.41,0.03)
1-3 visit	0.35(-0.34,1.05)	-0.80(-1.47,-0.13)*
**Party**		
Multipara	0.37(-0.15,0.88)	0.46(-1.08,1.99)
**Haemorrhage**		
Yes	1.34(0.66,2.02)	0.95(0.19,1.71)*
**Premature rupture of membrane**		
Yes	1.08(0.58,1.58)	0.23(-0.42,0.89)
**Presentation**		

				Non-vertex© 1.56(1.06,2.05) 1.19(0.64,1.74)*<br>**Gestational age**<br><37 week 1.94(1.45,2.43) 1.17(0.47,1.88)*<br>**Birth weight**<br><2500g 2.22(1.73,2.72) 0.73(0.01,1.45)*<br>**Sex of the neonate**<br>Male 1.47(0.82,2.12) 0.90(0.19,1.60)*<br>*©breech, transverse, face and brow, NA: not applicable and *Significant at P<0.05*
		(*b*) Report category boundaries when continuous variables were categorized		Not applicable
		(*c*) If relevant, consider translating estimates of relative risk into absolute risk for a meaningful time period		Not applicable
Other analyses	17	Report other analyses done—eg analyses of subgroups and interactions, and sensitivity analyses	-	Not applicable
**Discussion**				
Key results	18	Summarise key results with reference to study objectives	7 and 8	In this study neonatal mortality incidence ratio were 9.6(95%CI: 7.5, 12.2) per 1000 live births. Age of the mother, number of ANC visit, haemorrhage, non-vertex presentation, gestational age, birth weight, and sex of the neonate had significant risk factor for neonatal mortality. The major causes of neonatal mortality were prematurity, infection, and birth asphyxia.<br>The incidence of neonatal mortality was lower than studies done in northern Ethiopia (18.6 per 1000 live births), Kersa Health and Demographic Surveillance system site in Ethiopia (27.5 per 1000 live births) and two studies in southwest Ethiopia (35.5 and 27 per 1000 live births). But, it was higher than one study done in South Central Ethiopia (4.8 per 1000 live births) [10, 12-15]. The reason for this is the study period difference along with advance in the health care system that people's attitudes and awareness about conditions that put the newborn for ill health and increase in health-seeking behavior from time to time. The causes of neonatal mortality (prematurity, infection, birth asphyxia, and anomalies) in this study were in line with different studies done in Ethiopia [10, 13, 14, 17].<br>Advanced maternal age (age greater than or equal to 35 years old) had a significant risk factor for neonatal mortality as identified in this study. This is the fact that advanced maternal age increases the risk that predisposes for different complications for the fetus, and for the neonates as well as for the mother. As indicated in this study, hemorrhage, and non-vertex presentation was a determinate factor for neonatal mortality. This was in line with studies done in

				Southeast Brazil, South Africa, Uganda, and two studies in Ethiopia [10, 11, 14, 22, 23]. The reason for this is that those stated conditions are the ones or in another way can affect the neonate during intra-uterine as well as extra-uterine life and predispose to life-threatening even for loss of life during the neonatal period.<br>A number of the ANC follow up had significant risk for neonatal mortality as point out in this study. This is congruent with studies done in Southeast Brazil, and three studies in Ethiopia [10, 14, 17, 22]. This is obvious that the pregnant mother avoids preventable risk factors after having several ANC follow up, early identification and treatment of pre-existing conditions, and early screening of conditions that occur during pregnancy. In this study, gestational age less than 37 weeks, and birth weight less than 2500g were the most determinate factor for neonatal mortality. This was consistent with the study done in Ethiopia [10]. This is because those newborns whose gestation age less than 37 weeks (preterm) and birth weight less than 2500g were more likely to develop different complications during and after delivery and results for severe morbidity and mortality.<br>Being a male neonate was a significant risk for neonatal mortality as showed in this study. This is in line with some of the studies done in Ethiopia [14, 16, 17]. This is maybe due to the nature that male neonates more risk for different complication as stated in many studies.<br>The public health importance of this study is: Neonates are the risk population group for different complications and most likely affected by preventable causes of morbidity and mortality. Nowadays the neonatal mortality is on the way of decreasing but it is not that much satisfactory as compared to under-five child mortality. So, studies on risk factors that predispose the newborn for ill health and mortality are very important to prevent the underlining causes and to give immediate solutions.
Limitations	19	Discuss limitations of the study, taking into account sources of potential bias or imprecision. Discuss both direction and magnitude of any potential bias	9	The limitations are: response of the verbal autopsy was written based on the respondent’s view and some of the causes were difficult to classify in one category. Besides, during follow up some mothers did not come to health care institutions for immunization as well as for other services and very challenged to trace those mothers as they were out of health facilities.
Interpretation	20	Give a cautious overall interpretation of results considering objectives, limitations, multiplicity of analyses, results from similar studies, and other relevant evidence	9	The results were interpreted with some cautions as per limitations of the study
Generalisability	21	Discuss the generalisability (external validity) of the study results	9	The main strength of this study that the design was prospective follow up that it gave a true measure of the incidence of neonatal mortality and to develop cause and effect relationship. Standard and validated verbal autopsy tool was used to measure the causes for neonatal mortality to maintain the validity and reliability. The large sample size was used for this study that resulted in high power and greater precision.

**Other information**				
Funding	22	Give the source of funding and the role of the funders for the present study and, if applicable, for the original study on which the present article is based	11	Arba Minch University provided funds for the data collection and stationary materials of this research work with a project grant code of GOV/AMU/TH.1.1/CMHS/NUR/04/10. The website of the university is www.amu.edu.et. "The funders had no role in study design, data collection and analysis, decision to publish, or preparation of the manuscript."
